# Synthesis, characterization and NLO properties of 1,4-phenylenediamine-based Schiff bases: a combined theoretical and experimental approach†

**DOI:** 10.1039/d3ra07642c

**Published:** 2024-01-30

**Authors:** Muhammad Tahir, Hina Aftab, Iqra Shafiq, Muhammad Khalid, Saadia Haq, Attalla F. El-kott, Mohamed Abdellatif Zein, Umme Hani, Zahid Shafiq

**Affiliations:** a Institute of Chemical Sciences, Bahauddin Zakariya University Multan 60800 Pakistan zahidshafiq@bzu.edu.pk; b Institute of Chemistry, Khwaja Fareed University of Engineering & Information Technology Rahim Yar Khan 64200 Pakistan khalid@iq.usp.br muhammad.khalid@kfueit.edu.pk; c Centre for Theoretical and Computational Research, Khwaja Fareed University of Engineering & Information Technology Rahim Yar Khan 64200 Pakistan; d Biology Department, College of Science, King Khalid University Abha Saudi Arabia; e Zoology Department, College of Science, Damanhour University Egypt; f Chemistry Department, University College of AlWajh, University of Tabuk Tabuk Saudi Arabia; g Chemistry Department, Faculty of Science, Damanhour University Egypt

## Abstract

In the current study, three novel 1,4-phenylenediamine-based chromophores (3a–3c) were synthesized and characterized and then their nonlinear optical (NLO) characteristics were explored theoretically. The characterization was done by spectroscopic analysis, *i.e.* FT-IR, UV-Visible, and NMR spectroscopy, and elemental analysis. Notably, these chromophores exhibited UV-Visible absorption within the range of 378.635–384.757 nm in acetonitrile solvent. Additionally, the FMO findings for 3a–3c revealed the narrowest band gap (4.129 eV) for 3c. The GRPs for these chromophores were derived from HOMO–LUMO energy values, which showed correspondence with FMO results by depicting a minimum hardness (2.065 eV) for 3c. Among these compounds, 3c displayed the highest nonlinear behavior with maximum *μ*_tot_, *β*_tot_ and *γ*_tot_ values of 4.79 D, 8.00 × 10^−30^ and 8.13 × 10^−34^ a.u., respectively. Our findings disclosed that the synthesized 1,4-phenylenediamine chromophores may be considered promising candidates for nonlinear optical materials, showing potential applications in the realm of optoelectronic devices.

## Abstract 2

In the current study, three novel 1,4-phenylenediamine-based chromophores (3a–3c) were synthesized and characterized and then their nonlinear optical (NLO) characteristics were explored theoretically.

## Graphical abstract

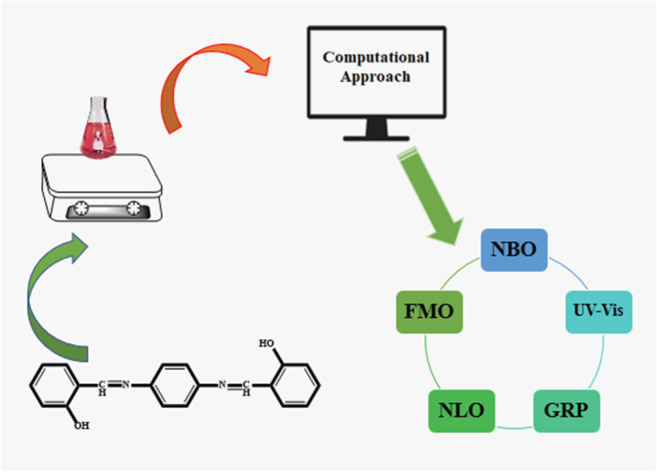

## 1. Introduction

Schiff bases are vital chemical compounds in the fields of organic, nonliving, analytical and therapeutic chemistry due to their flexibility^1^ and structural diversity. Schiff bases can be produced by the reaction of a carbonyl group of either ketone or aldehyde with primary amines.^2^ Schiff bases are also called azomethines or imines owing to the presence of a C=N chromophore.^3^ Different analogues of Schiff bases have been used in normal life sciences due to their anticancer,^4^ antimicrobial,^5,6^ antimalarial,^7^ antifungal,^8^ antibacterial^9^ and antioxidant^10^ potential. Moreover, they serve as a catalyst for different reactions like the reduction of thionyl chloride,^11^ an aldol condensation reaction,^12^ the hydrogenation of olefins,^13^ the ability to bind oxygen in epoxidation reactions,^14^ and photochromic properties,^15^ as well as acting as corrosion inhibitors for various metals like steel, aluminum and copper.^16^ Additionally, they can be employed as chelating agents in foodstuffs.^17^ Schiff bases play an important role as an NLO active material due to their property of solvatochromicity.^18,19^ The innovations of solid-phase extraction techniques^20^ and ion-selective electrodes for identifying anions in analytical experiments could both benefit from these ligands.^21^

The aim of NLO is to describe the reaction to an applied electric field.^22,23^ Electro-optics for signal processing and frequency variations,^24^ particularly in telecommunications,^25^ information technology and fiber optics, benefit greatly from the use of NLO compounds.^21,26^ They tend to allow structural alterations to enable a range of NLO behaviors due to their simple chemistry and inexpensive cost of production.^24^ Recent research has focused on NLO materials established from organic networks.^27^ Intramolecular charge transfer (ICT) from donating (D) to accepting (A) moieties of electrons *via* conjugation is a valuable property of NLO organic materials.^28–30^ Computational and experimental data indicate that a broad second-order NLO response can be managed by introducing strong D and A groups on the spacer's paradoxical sides, *i.e.*, A–D–A, D–D–A and D–A.^31^ Charge transfer can be improved by compounds with delocalized electrons in a D–A configuration.^32^ When subjected to intense laser light, NLO materials are responsible for a broad range of applicability due to their exciting photo-physical behavior.^33^ There is a lot of interest in improving synthetic organic compounds to have a quick response rate, a better laser impairment approach, a higher photo-electrical quantum yield,^34^ a small dielectric constant,^35^ construction flexibility, and low improvement cost.^36,37^ This report focuses on creating various new 1,4-phenylenebis(azaneylylidene)bis(methaneylylidene)-based compounds. The characterization of these compounds was accomplished by employing a variety of spectroscopic techniques. The DFT method was utilized to thoroughly investigate their optoelectronic properties.

## 2. Experimental section

### 2.1 Chemistry

All manipulations were carried out with chemicals from Sigma-Aldrich in pure form. Elemental analyses were performed on a PerkinElmer 2400 Combustion CHN Analyzer. IR spectra on solid samples were obtained using an FT-IR IFS48 spectrophotometer. The NMR spectra were obtained in DMSO-d_6_ solvent using a Bruker AVANCE III HD 400 MHz instrument. The NMR spectra are given in terms of tetramethyl silane (=0 ppm). The progress and completion of the reaction were monitored by TLC.

### 2.2 General procedure for the synthesis of *ρ*-phenylenediamine derivatives

Schiff bases 3a–3c were prepared by the condensation reaction of *ρ*-phenylenediamine with corresponding aromatic aldehydes and ketones in a molar ratio of 1 : 2. This synthesis was performed by adding *ρ*-phenylenediamine (1) (0.2 g, 0.002 mmol) to the respective ketone or aldehyde (2a–c) (0.004 mmol), refluxed in 15 ml of hot ethanol with a catalytic amount of acetic acid (CH_3_COOH) for 5 to 15 hours at 70 to 80 °C. The solid precipitates were separated through filtration, rinsed with both warm and cold ethanol and ultimately air-dried at room temperature. The resulting colored products 3a–3c were obtained in good yield. These compounds have also been reported elsewhere^38–40^ for other applications.

The targeted compounds were characterized as follows.

#### 2.2.1 2,2′-((1*E*,1′*E*)-(1,4-phenylenebis(azanylylidene))bis(methanylylidene))diphenol (3a)

Yield 85%, m.p. 219–220 °C; (UV-Vis): *λ*_max_ = 366 nm (acetonitrile), 364 nm (methanol); FT-IR (cm^−1^): 1568 cm^−1^ (C=N), 3050 cm^−1^ (OH); ^1^H NMR (400 MHz, DMSO) *δ* 13.07 (s, 1H), 9.04 (s, 1H), 7.68 (dd, *J* = 7.7, 1.6 Hz, 1H), 7.55 (s, 2H), 7.43 (ddd, *J* = 8.3, 7.4, 1.7 Hz, 1H), 7.00 (ddd, *J* = 10.9, 6.0, 2.2 Hz, 2H). ^13^C NMR (101 MHz, DMSO) *δ* 163.64, 160.77, 147.16, 133.83, 133.05, 123.04, 119.85, 119.69, 117.10: Anal. calcd for C_20_H_16_N_2_O_2_ (316.35); C, 75.93; H, 5.10; N, 8.86; found; C, 75.89; H, 5.14; N, 8.96.

#### 2.2.2 6,6′-((1*E*,1′*E*)-(1,4-phenylenebis(azanylylidene))bis(methanylylidene))bis(2-ethoxyphenol) (3b)

Yield 73%, m.p. 189–190 °C; UV-Vis: *λ*_max_ = 416 (acetonitrile), 414 (methanol); FT-IR (cm^−1^): 1604 cm^−1^ (C=N), (OH); ^1^H NMR (400 MHz, DMSO) *δ* 13.27 (s, 1H), 9.03 (s, 1H), 7.55 (s, 2H), 7.25 (dd, *J* = 7.9, 1.4 Hz, 1H), 7.13 (dd, *J* = 8.0, 1.3 Hz, 1H), 6.91 (t, *J* = 7.9 Hz, 1H), 4.09 (q, *J* = 7.0 Hz, 2H), 1.36 (t, *J* = 7.0 Hz, 3H);^13^ NMR (101 MHz, DMSO) *δ* 163.85, 151.36, 147.52, 146.91, 124.60, 123.03, 119.84, 119.14, 117.54, 64.61, 15.27; Anal. calcd for C_24_H_24_N_2_O_4_ (404.46); C, 71.27; H, 5.98; N, 6.93; found; C, 71.23; H, 5.94; N, 6.99.

#### 2.2.3 4,4′-((1*E*,1′*E*)-(1,4-phenylenebis(azanylylidene))bis(methanylylidene))bis(benzene-1,3-diol) (3c)

Yield 80%: m.p. 259–260 °C; UV-Vis: *λ*_max_ = 374 nm (acetonitrile), 380 nm (methanol); FT-IR (cm^−1^): 1596 cm^−1^ (C=N), 3223 cm^−1^ (OH); ^1^H NMR (500 MHz, DMSO-d_6_) 13.53 (1H, s), 10.24 (1H, s), 8.83 (1H, s), 7.42 (3H, d, *J* 8.4), 6.40 (1H, dd, *J* 8.5, 2.3), 6.30 (1H, d, *J* 2.3); ^13^C NMR (126 MHz, DMSO) *δ* 163.20, 162.64, 162.15, 146.28, 134.56, 122.24, 112.31, 108.08, 102.56. Anal. calcd for C_20_H_16_N_2_O_4_ (348.35); C, 68.96; H, 4.63; N, 8.04; found; C, 68.92; H, 4.66; N, 8.09.

### 2.3 Computational procedure

The 1,4-phenylenediamine-based compounds (3a–3c) with A–π–A configuration were optimized with the M06/6-311G(d,p)^41^ functional. Then in all other investigations, NBO and NLO were carried out at the above-mentioned level. All calculations were conducted utilizing the Gaussian 09 (ref. 42) software, and the results were examined through Gauss view 5.0.^43^ The absorption spectra, FMOs and global reactivity descriptors were determined by the TD-DFT method with the aforesaid functional in acetonitrile. The findings from the output files were analyzed using PyMOlyze,^44^ Chemcraft,^45^ Avogadro,^46^ GaussSum,^47^ Origin 8.0 (ref. 48) and Multiwfn 3.7 (ref. 49) software. Moreover, *μ*_tot_,^50^ 〈*α*〉,^51^*β*_tot_^52^ and *γ*_tot_^53^ were calculated through eqn (1)–(4).

1 *μ* = (*μ*_*x*_^2^ + *μ*_*y*_^2^ + *μ*_*z*_^2^)^1/2^

2 〈*α*〉 = 1/3(*α*_*xx*_ + *α*_*yy*_ + *α*_*zz*_)

3 *β*_tot_ = [*β*_*x*_^2^ + *β*_*y*_^2^ + *β*_*z*_^2^]^1/2^

where, *β*_*x*_ = *β*_*xxx*_ + *β*_*xyy*_ + *β*_*xzz*_, *β*_*y*_ = *β*_*yyy*_ + *β*_*xxy*_ + *β*_*yzz*_ and *β*_*z*_ = *β*_*zzz*_ + *β*_*xxz*_ + *β*_*yyz*_.4
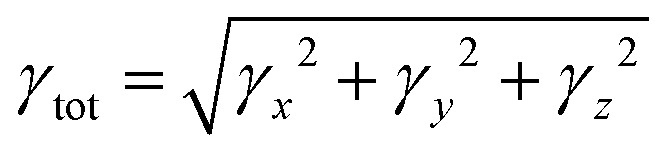
where, 
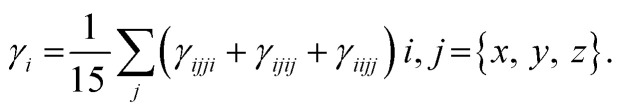


Global reactivity descriptors, such as global softness (*σ*), ionization potential (IP),^32^ global hardness (*η*),^31^ global electrophilicity index (*ω*),^33^ electronegativity (*X*),^35^ charge transfer rate (Δ*N*_max_), chemical potential (*μ*)^34^ and electron affinity (EA) were determined using eqn (5)–(12).

5 IP = −*E*_HOMO_

6 EA = −*E*_LUMO_

7
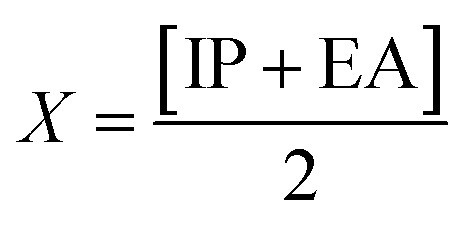

8 *η* = IP − EA

9
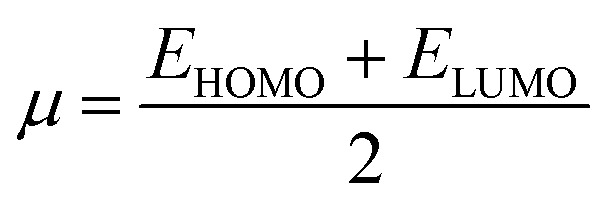
10
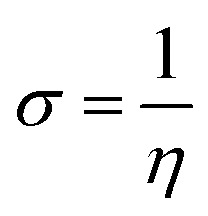
11
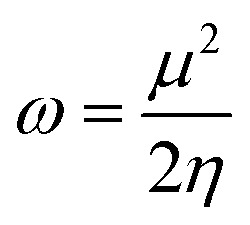
12
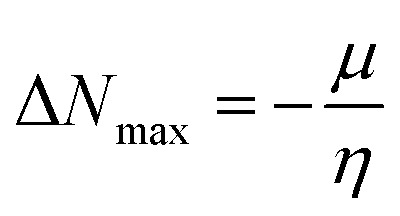


### 2.4 Results and discussion

**Scheme 1:**
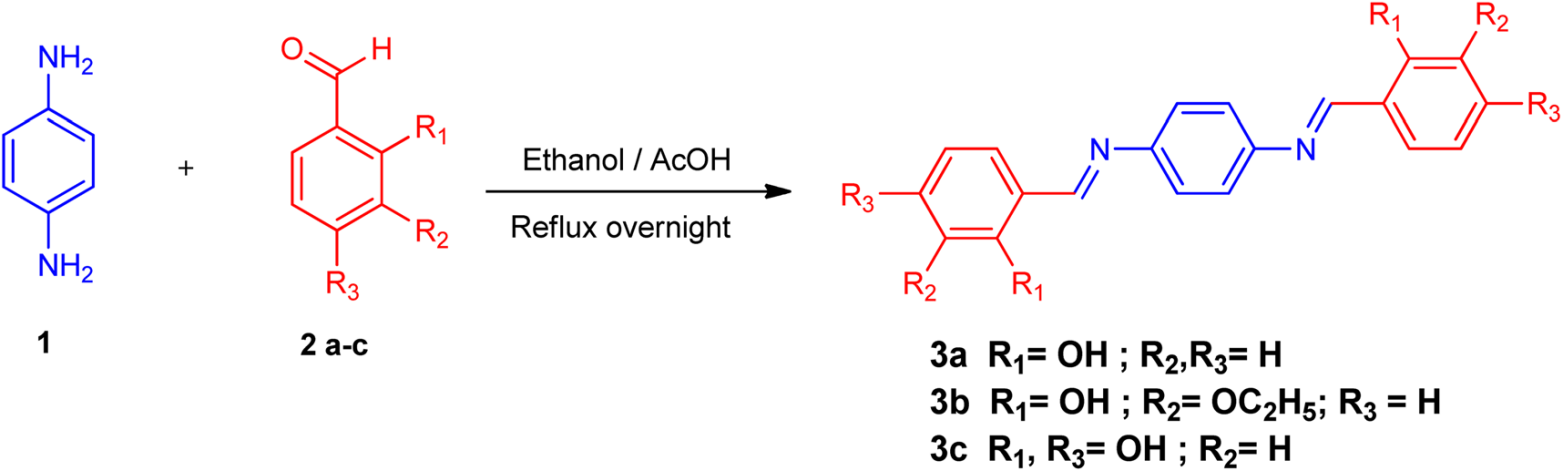
Synthesis of ρ-phenylenediamine derivatives.

**Scheme 2:**
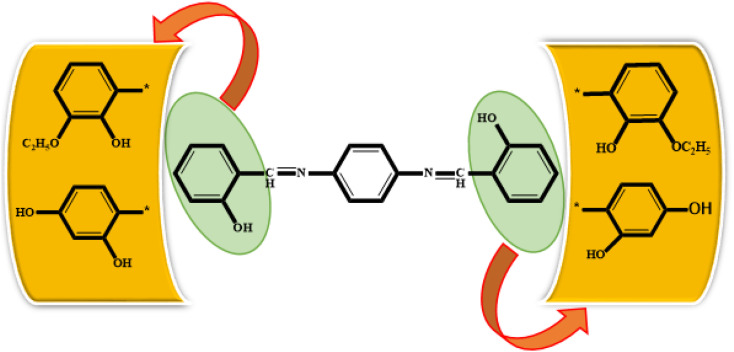
Schematic representation of 3a, 3b and 3c molecules.

All synthesized Schiff bases were characterized by employing elemental and spectral analyses *i.e.*, UV-Vis, FT-IR, ^1^H-NMR, and ^13^C-NMR spectroscopies (Fig. S1–S9†). Elemental analysis shows that the synthesized Schiff bases have (C, H, N) elements. UV-Vis absorption values were recorded in various solvents of different polarity, *i.e.*, MeCN and methanol, providing information about the nature of the transitions and charge transfer capabilities of the synthesized compounds.^54^ The characteristic π–π* transition in –C=N– was recorded. The value of the absorption is shifted bathochromically when the solvent polarity increases. The observed differences can be ascribed to an interaction involving hydrogen bonding between the molecules of the solute and the solvent. The FT-IR spectra provide information about the nature of the functional groups.^55,56^ The IR spectra of the synthesized compounds (3a–3c) show bands related to aromatic and aliphatic C–H stretching vibrations in the range 2835–3150 cm^−1^, while C–N and C–O stretching vibrations were detected in the range 1150–1630 cm^−1^. A sharp peak at 1568 cm^−1^ is due to (C=C) stretching vibration. A medium band corresponding to the phenolic oxygen (C–O) appears within the range 1275–1298 cm^−1^. The IR spectra of 3a–3c show a band in the region 2987–3300 cm^−1^ due to –OH stretching vibration. A band in the range 1568–1605 cm^−1^ is observed due to (C=N). ^1^H-NMR provides information about the resonance of protons based on their integration and multiplicity pattern. NMR spectroscopic measurements were made for 3a–c using TMS as the internal standard. In ^1^H-NMR, the –CH=N signal appeared as a singlet around *δ* 8.96–9.05 ppm, another singlet appeared due to the OH group at *δ* 12.75–13.25 ppm. It is important to highlight that phenolic protons consistently exhibit a singular peak at elevated *δ* values, indicating their interaction with an adjacent nitrogen atom. In the range 7.00–8.50 ppm, a multiplet appeared due to the protons of the benzene ring. The free NH_2_ protons usually exhibit a wide singlet peak within the 4–6 ppm range. However, in the Schiff base spectrum under consideration, this signal is notably absent, suggesting the creation of a Schiff base. In the ^13^C-NMR spectra of 3a–3c in DMSO, the signal peak of the carbon atom is found at 155.7 ppm, indicating a double-bond character, whereas in the corresponding spectra of the synthesized Schiff bases the signal peak appears at 163.19 ppm, indicating C=N character. After the structural confirmation of 3a–3c, quantum chemical calculations were accomplished with the M06/6-311G(d,p) functional of the DFT approach. The IUPAC names of the studied chromophores with A–π–A framework are: 2,2′-((1,4-phenylenebis(azaneylylidene))bis(methaneylylidene))diphenol (3a), 6,6′-((1*E*,1′*E*)-(1,4-phenylenebis(azaneylylidene))bis(methaneylylidene))bis(2-ethoxyphenol) (3b) and 4,4′-((1*E*,1′*E*)-(1,4-phenylenebis(azaneylylidene))bis(methaneylylidene))bis(benzene-1,3-diol) (3c). The end-capped acceptor moieties played a substantial part in achieving effective NLO-based chromophores.^57^ The structures of the titled compounds (3a–3c) are displayed in Fig. S10† and Scheme 1. Accompanying this, their Cartesian coordinates are presented in Tables S11–S13.† TD-DFT/DFT calculations were conducted in order to evaluate the influence of different acceptor variants on intra-molecular charge transfer, band gap, nonlinear response and absorption spectra (Scheme 2).

### 2.5 Frontier molecular orbital (FMO) analysis

FMOs serve as crucial descriptors, collectively characterizing a molecule's optical and electronic properties, as well as its chemical reactivity.^58–60^ The HOMO and LUMO offer insights into the energy difference between the lowest energy state and the next higher energy state.^33,61–63^Table 1 provides the HOMO and LUMO energy values, along with their respective energy differences. Further outcomes are illustrated in Table S1.†

**Table 1:** Energies (eV) of frontier molecular orbitals for 3a–3c

Compound	*E*_HOMO_	*E*_LUMO_	Δ*E*
3a	−6.098	−1.965	4.133
3b	−6.090	−1.907	4.183
3c	−5.892	−1.763	4.129

**Fig. 1:**
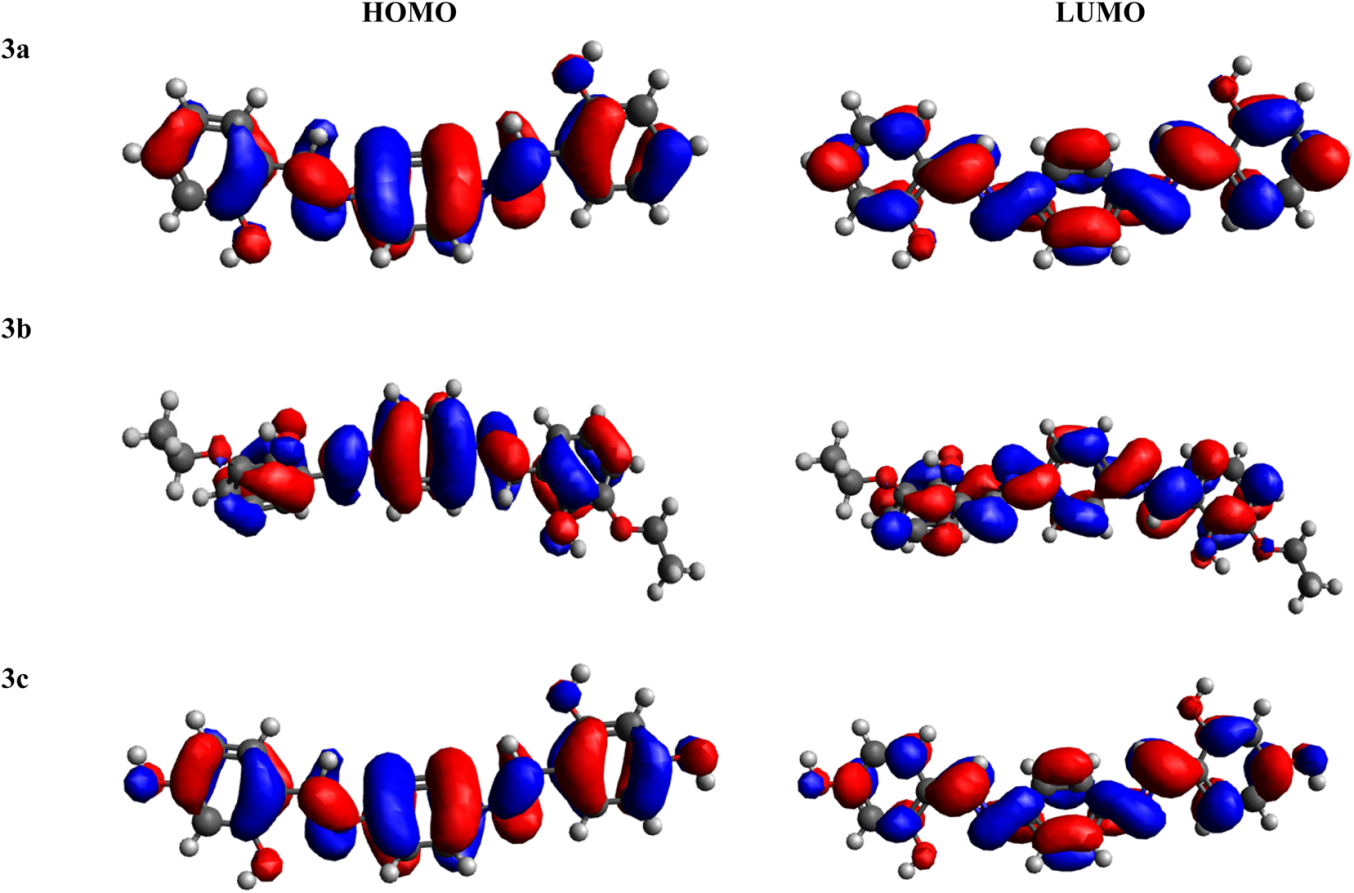
HOMO–LUMO pictographic representation of 3a–3c.

Amongst all of the examined chromophores (3a–3c), compound 3c exhibited the smallest HOMO–LUMO energy difference, measured at 4.129 eV, along with the smallest HOMO and LUMO energy levels at −5.892 and −1.763 eV, respectively. This phenomenon highlights a proficient charge transfer characteristic within this compound. The minimal band gap observed could be ascribed to the existence of two strongly electronegative –OH units over the acceptor portion, generating a powerful push–pull effect. In contrast, the greatest band gap is identified in compound 3b, measuring 4.183 eV, featuring the greatest energy LUMO/LUMO at −1.907/−6.090 eV, respectively. This maximum Δ*E* resulted from the presence of an ethoxy group at both ends of the acceptor moiety. The HOMO energy value for 3a is −6.098 eV, while for LUMO, the energy value is −1.965 eV. The decreasing order of Δ*E* for all the chromophores is 3b > 3a > 3c. In addition to analyzing energy levels, the study of charge transfer between orbitals is another essential aspect of FMO study. Analysis of the MO surfaces of the investigated chromophores (Fig. 1 and S11†) indicates whole transfer of the charge throughout the entire molecular structure. This observation illustrates the efficient intra-molecular CT that takes place throughout the entire molecule, both in the HOMO and LUMO orbitals of all the studied chromophores. In summary, all the compounds under investigation exhibited a comparable bandgap. Nevertheless, 3c exhibited a relatively lower band gap (4.129 eV), indicating greater charge transfer, higher polarizability and red shift spectra within this chromophore, which as a result made it effective as an NLO material.

### 2.6 Global reactivity parameters (GRPs)

The global reactivity descriptors represent insights into the chemical reactivity and stability of chromophores.^64,65^

IP signifies the energy needed to remove an electron from a molecular orbital. From Table 2, it is evident that the OH groups at both ends of the acceptor unit of 3c reduced the IP. The decreasing direction of IP values for 3a–3c is noted as 3a > 3b > 3c at 6.098, 6.090 and 5.892 eV, respectively. Conversely, electron affinity (EA) pertains to the energy released when an electron is added into a valence shell to form an anion. Consequently, these descriptors find utility in gaining insights into molecular stability. 3c represented the smallest EA at 1.763 eV, while 3a at 1.965 eV showed the highest EA. Information about electronic cloud polarization can be effectively obtained with *σ* and *η*. Hard compounds demonstrate a higher level of stability due to their weak electron polarization density, while reactive soft molecules display increased electronic cloud polarization. Among them all, 3b emerged as the hardest with an *η* value of 2.092 eV, while 3a is identified as the *σ* molecule, featuring a value of 0.243 eV^−1^. Consequently, 3a demonstrated amplified electronic cloud polarization, indicative of a strong NLO response. It should be noted that the parameters *μ* and *X* exhibit an inverse relationship with each other.^41^ In the case of 3a, it possesses the highest *X* and the lowest *μ* values, recorded at 4.032 and −4.032 eV, respectively (as shown in Table 2). These results signified the greater charge transference and in turn, improved the NLO response in these chromophores.

**Table 2:** Global reactivity descriptors of studied compounds (3a–3c)^a^

Compound	IP	EA	*X*	*η*	*H*	*ω*	*σ*	Δ*N*_max_
3a	6.098	1.965	4.032	2.067	−4.032	3.932	0.243	1.951
3b	6.090	1.907	3.999	2.092	−3.999	3.822	0.239	1.912
3c	5.892	1.763	3.828	2.065	−3.828	3.548	0.242	1.853

a Unit = eV, *σ* is in eV^−1^.

### 2.7 Natural bond orbital (NBO) investigation

NBO investigation offers insights into the structural aspects of chromophores, elucidating the characteristics of valence orbitals, localized bonds, and anti-bonds. Furthermore, this examination reveals the stabilizing interactions present in both filled and vacant orbitals.^66,67^ The Δ*E* between interacting orbitals governs the extent of stabilization in these orbital interactions. Consequently, effective D and A groups typically exhibit the highest stabilizing interactions. Table 3 displays the most significant energy transitions, including σ → σ*, π → π*, LP → σ* and LP → π, with the highest and lowest energy values. The remaining data is presented in Tables S2–S4.†

**Table 3:** Interpretation of Fock matrix results using second-order perturbation theory for 3a–3c

Compound	Donor (*i*)	Type	Acceptor (*j*)	Type	*E*(2) [kcal mol^−1^]	*E*(*j*) − *E*(*i*) [a.u.]	*F*(*i*, *j*) [a.u.]
3a	C29–C32	π	C26–C27	π*	26.25	0.29	0.081
	C26–C27	π	C26–C27	π*	1.95	0.29	0.021
	C19–C22	σ	C17–C19	σ*	5.29	1.29	0.074
	C19–C22	σ	O35–H36	σ*	0.52	1.14	0.022
	O37	LP(2)	C26–C27	π*	33.73	0.37	0.107
	N12	LP(1)	C13–H14	σ*	13.09	0.72	0.088
3b	C2–C3	π	C4–C51	π*	9.85	0.3	0.07
	C1–C6	π	C4–C52	π*	0.02	0.3	0.071
	C26–C28	σ	C28–C31	σ*	5.1	1.3	0.073
	C26–C28	σ	C26–O35	σ*	0.5	1.09	0.021
	O40	LP(2)	C22–C23	π*	9.82	0.37	0.1
	O40	LP(1)	C22–C23	σ*	7.19	1.16	0.082
3c	C1–C6	π	C2–C3	π*	22.34	0.3	0.073
	C28–C30	π	C28–C30	π*	2.18	0.29	0.023
	O41–H42	σ	C27–C30	σ*	4.81	1.33	0.072
	C28–H36	σ	C28–C30	σ*	0.62	1.09	0.023
	O33	LP(2)	C25–C27	π*	35.86	0.37	0.109
	N11	LP(1)	C1–C6	σ*	6.99	0.93	0.073

The interactions (Table 3) are primarily centered on the overlap of π(C–C) → π*(C–C) orbitals within the aromatic rings, facilitating ICT and ultimately leading to stabilization, which in turn, leads to the weakening of bonds because of increased electron density within the C–C anti-bonding orbitals.

In 3a, the most substantial stabilization energy arising from π to π* transitions among all the compounds is identified as 26.25 kcal mol^−1^, specifically for the orbitals π(C29–C32) to π*(C26–C27). The lowest stabilizing energy is observed in the interaction between π orbitals of carbon atoms C26 and C27, specifically the transition from π to π* with a value of 1.95 kcal mol^−1^. In the context of transitions from σ to σ* orbitals, the maximum stabilization energy of 5.29 kcal mol^−1^ occurs in compound 3a. This transition involves the orbitals σ(C19–C22) to σ*(C17–C19). On the other hand, the minimum energy value, 0.52 kcal mol^−1^, is found in the transition from σ(C19–C22) to σ*(O35–H36). The lone pair undergoes transitions with the highest and lowest energy values at 33.73 and 13.09 kcal mol^−1^ for the LP(2)(O37) to π*(C26–C27) and LP(1)(N12) to σ*(C13–H14) transitions, respectively.

In the case of 3b, the π to π* transitions exhibit a high stabilization energy of 9.85 kcal mol^−1^, observed in the transition from π(C2–C3) to π*(C4–C51). Conversely, the interaction involving orbitals of π(C1–C6) to π*(C4–C52) shows the minimum value of 0.02 kcal mol^−1^. For σ to σ* interaction, the highest and lowest values are 5.1 and 0.5 kcal mol^−1^, respectively. These values are associated with transitions from σ(C26–C28) to σ*(C28–C31) and σ(C26–C28) to σ*(C26–O35). Regarding lone-pair transitions, 3b exhibits the highest energy transition of 9.82 kcal mol^−1^ from LP(2)(O40) to π*(C22–C23) and the lowest energy transition of 7.19 kcal mol^−1^ from LP(1)(O40) to σ*(C22–C23).

Similarly, in the case of 3c, the highest value of stabilization energy is 22.34 kcal mol^−1^ for the transition from π(C1–C6) to π*(C2–C3). On the other hand, the lowest value, 2.18 kcal mol^−1^, is found in transitions linking orbitals of π(C28–C30) to π*(C28–C30). Regarding σ to σ* transitions, there are greater and smaller values of 4.81 and 0.62 kcal mol^−1^ in σ(O41–H42) to σ*(C27–C30) and σ(C28–H36) to σ*(C28–C30), respectively. Lone-pair transitions in 3c, such as LP(2)(O33) to π*(C25–C27) and LP(1)(N11) to σ*(C1–C6), exhibit values of 35.86 and 6.99 kcal mol^−1^, respectively. These elevated stable energy values indicate significant hyper-conjugation, leading to improved CT and ultimately contributing to the nonlinearity of the studied chromophores.

### 2.8 UV-Visible analysis

**Fig. 2:**
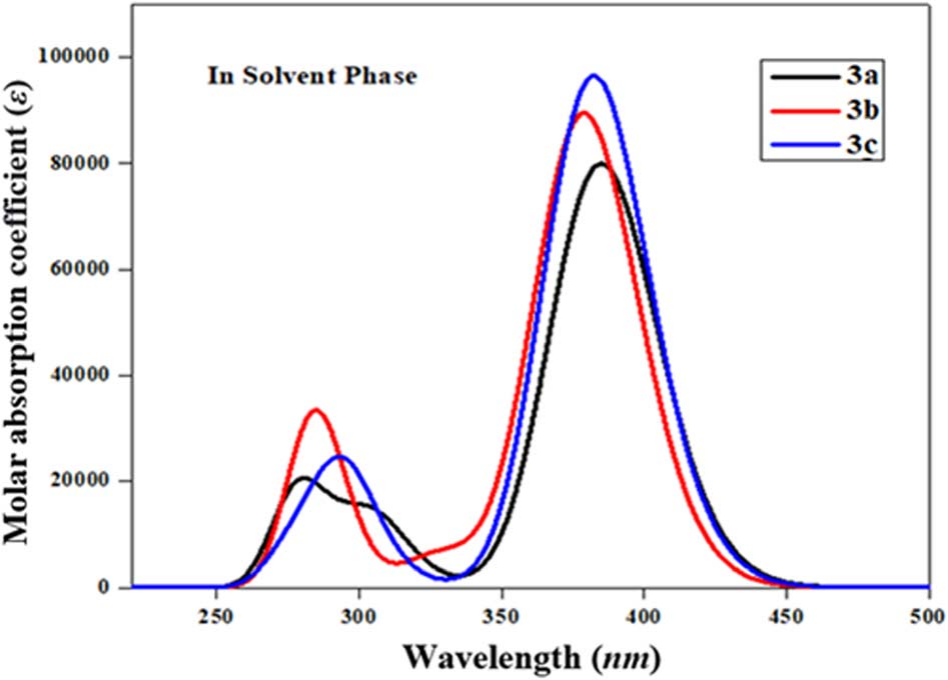
The computed UV-Vis spectra of 3a–3c in acetonitrile.

The UV-Vis spectral analysis of 3a–3c was conducted in acetonitrile solvent to elucidate significant electronic transitions, including excitation energy and oscillator strength.^67,68^Tables 4 and S5–S7† provide essential data, such as absorption maxima (*λ*_max_), excitation energy (*E*), oscillator strength (*f*_os_), and details regarding the contributions of molecular orbitals. Electronic absorption is chiefly determined by the transition from the ground to the excited state, which is predominantly characterized by the electronic excitations from the HOMO to the LUMO.^69^ The absorption bands of these studied chromophores (3a–3c) are visually represented in Fig. 2.

**Table 4:** Wavelength (*λ*_max_), excitation energy (*E*), oscillator strength (*f*_os_) and nature of molecular orbital contributions of compounds 3a–3c in the solvent phase^a^

Compounds	*λ* (nm)	*E* (eV)	*f*_os_	MO contributions
3a	384.757	3.222	1.104	H → L (94%)
3b	378.635	3.275	1.234	H → L (93%)
3c	382.172	3.244	1.332	H → L (94%)

a Solvent = acetonitrile, MO = molecular orbital, H = HOMO, L = LUMO, *f*_os_ = oscillator strength, wavelength = *λ* (nm).

Table 4 shows that amongst all the studied compounds (3a–3c), 3a displayed the highest absorption peak at 384.757 nm, accompanied by a maximum oscillator strength (*f*_os_) of 1.104 and excitation energy (*E*) of 3.222 eV. Remarkably, this electronic transition from the HOMO to the LUMO accounted for a substantial 94% contribution. It is noteworthy that this absorption wavelength (*λ*_max_) aligned with the lower band gap observed in 3a, as evident from the FMO results. This might be due to the presence of an alcoholic group (–OH) in the end-capped acceptor moiety. The maximum wavelength values of 3c and 3b compounds are observed at 382.172 and 378.635 nm accompanied by 1.332 and 1.234 *f*_os_, respectively, while their *E* values are observed to be 3.244 and 3.275 eV, respectively (Table S5†). These *λ*_max_ for the abovementioned compounds involved transitions with 94 and 93% contributions from HOMO to LUMO, respectively. These values correspond to the presence of two –OH groups in the acceptor of 3c and the 2-ethoxy-6-methylphenol group in 3b. The decreasing order of maximum absorption wavelength of the studied chromophores based on highest *f*_os_ in the solvent phase is found to be: 3a > 3c > 3b. Recalling the NBO results (Table 3), it becomes apparent that the molecular orbitals primarily contributed to π → π* and n → π* interactions in the 3a–3c. It is worth noting that the absorption maxima (*λ*_max_) can be significantly influenced by the polarity and characteristics of the solvent, a phenomenon well demonstrated in previous literature.^70^

### 2.9 Non-linear optical (NLO) analysis

NLO effects result from the interaction of electromagnetic (EM) forces with different materials. This interaction gives rise to the production of novel EM waves that exhibit changes in frequency, phase, or amplitude, and display unique propagation characteristics when compared to the original incident wave.^71^ The link between geometry and non-linear optical effects is characterized by properties like charge transfer and hyper-polarizability.^72^ Materials exhibiting a strong NLO response tend to possess large dipole moments, linear polarization and substantial hyper-polarizability values. The linear polarizability (*α*) parameter characterizes the ability of an electric field to distort the electronic distribution within a molecule. Conversely, hyper-polarizabilities (*β*, *γ etc.*) represent the NLO response originating from molecules and atoms, encompassing the comprehensive phenomena of nonlinear optics. The calculation of *β* is accomplished using the finite field method. In the presence of an applied electric field, the system's energy becomes a function of that electric field. The computed values of *μ*_tot_, 〈*α*〉, *β*_tot_, *γ*_tot_ and their respective tensors for 3a–3c are presented in Tables S8–S11,† with the key findings summarized in Table 5.

**Table 5:** Computed linear and nonlinear characteristics of 3a–3c a

Compound	*μ*_tot_	〈*α*〉 × 10^−23^	*β*_tot_ × 10^−30^	*γ*_tot_ × 10^−34^
3a	4.47	5.83	1.40	7.28
3b	4.26	6.99	1.23	7.42
3c	4.79	6.16	8.00	8.13

a *μ*_tot_ = Debye while other properties are in a.u.

The maximum value of the dipole moment (4.79 D) is displayed by 3c, which might be due to its lowest HOMO/LUMO band gap, whereas the minimum value (4.26 D) is shown by 3b. The trend for *μ*_tot_ values in 3a–3c is as follows: 3b < 3a < 3c. This is compared to the *μ*_tot_ value of standard *para*-nitroaniline (*p*-NA), which is 4.9662 D.^41^3a–3c exhibited comparable results to that of *p*-NA. The presence of highly electronegative atoms tends to attract charge density towards themselves, thereby contributing to enhanced linear polarizability. The observed average polarizability trend for 3a–3c is: 3a < 3c < 3b. In the case of 3b, the major contributing tensor is along the *x*-axis for 〈*α*〉, making it the primary contributor to the overall value. When comparing 〈*α*〉 with that of *p*-NA (1.17 × 10^−23^ a.u.), it is found to be 4.98 times higher in 3a, 5.97 times higher in 3b and 5.26 times higher in 3c, depicting their significantly elevated linear polarizabilities.

The hyper-polarizability of molecules typically relies on the potency of substituents attached to the acceptor group, such as hydroxyl, ethyl, or methyl groups. These substituents play a role in enhancing molecular nonlinearity. Additionally, extended conjugation within molecules also plays a significant role in hyper-polarizability, and this can be altered by substituents. Among all the compounds, 3c exhibited the highest *β*_tot_ value *i.e.*, 8.00 × 10^−30^ a.u. On the other hand, the presence of hydroxyl as well as ethoxy groups in 3b resulted in the minimum intra-molecular charge transfer and consequently, the lowest *β*_tot_ value (1.23 × 10^−30^ a.u.). The arrangement of *β*_tot_ values in ascending order for 3a–3c is determined as follows: 3b has a lower value than 3a, and 3a has a lower value than 3c. Furthermore, when comparing the *β*_tot_ results of *p*-NA (3.61 × 10^−31^ a.u.)^41^ with our studied compounds, significantly higher values are observed in 3a–3c. These findings highlight the enhanced *β*_tot_ of our investigated compounds, rendering them suitable candidates for innovative NLO materials. Moreover, amongst all the compounds, 3c also depicted the highest value for *γ*_tot_, which is found at 8.13 × 10^−34^ a.u. Whereas the lowest value of *γ*_tot_, *i.e.*, 7.28 × 10^−34^ a.u., is observed in 3a. The *γ*_tot_ value of standard *p*-NA corresponds to 2.71 × 10^−36^ a.u., which is smaller than the values for 3a–3c. Notably, the order of *γ*_tot_ of all the titled molecules can be written as follows: 3a < 3b < 3c. In conclusion, it is evident that all the aforementioned compounds hold potential as NLO materials. Amongst them all, 3c emerged as an exceptional candidate for nonlinear optics due to its outstanding optical properties.

## 3. Conclusion

In the current study, 1,4-phenylenediamine-based chromophores were synthesized and then their structural confirmation was accomplished through different spectroscopic techniques. Accompanying this, DFT calculations were also performed to determine the optoelectronic properties of 3a–3c chromophores. FMO analysis demonstrated the significant charge transfer in 3c with a narrowed bandgap (4.129 eV) and lower hardness (*η* = 2.065 eV) among 3a–3c. Furthermore, NBO investigation also supported the significant CT in all the studied compounds, attributed to hyper-conjugative interactions within the bonds, which contribute to molecular stability and the dispersion of electrons throughout the entire system. Among all the derivatives, 3a notably exhibited the most significant bathochromic shift (*λ*_max_ = 384.575 nm) accompanied by an oscillator strength (*f*_os_) of 1.104 with a transition energy of 3.222 eV in acetonitrile media. All the synthesized Schiff bases exhibited a significant NLO response compared with *p*-NA. Among 3a–3c chromophores, 3c exhibited notably first-order and second-order hyper-polarizabilities: 8.00 × 10^−30^ and 8.13 × 10^−34^ a.u., respectively. The above findings disclosed that all the synthesized Schiff bases, particularly 3c, exhibited an efficient NLO response and could be utilized as an efficient photonic material.

## Conflicts of interest

There are no conflicts to declare.

## Supplementary Material

RA-014-D3RA07642C-s001

## References

[cit1] Alorini T. A., Al-Hakimi A. N., Saeed S. E.-S., Alhamzi E. H. L., Albadri A. E. (2022). Arabian J. Chem..

[cit2] Rauf A., Shah A., Munawar K. S., Khan A. A., Abbasi R., Yameen M. A., Khan A. M., Khan A. R., Qureshi I. Z., Kraatz H.-B. (2017). J. Mol. Struct..

[cit3] Segura J. L., Mancheño M. J., Zamora F. (2016). Chem. Soc. Rev..

[cit4] Noser A. A., Abdelmonsef A. H., El-Naggar M., Salem M. M. (2021). Molecules.

[cit5] Shi L., Ge H.-M., Tan S.-H., Li H.-Q., Song Y.-C., Zhu H.-L., Tan R.-X. (2007). Eur. J. Med. Chem..

[cit6] Matar S. A., Talib W. H., Mustafa M. S., Mubarak M. S., AlDamen M. A. (2015). Arabian J. Chem..

[cit7] Tople M. S., Patel N. B., Patel P. P., Purohit A. C., Ahmad I., Patel H. (2023). J. Mol. Struct..

[cit8] Mi Y., Chen Y., Tan W., Zhang J., Li Q., Guo Z. (2022). Carbohydr. Polym..

[cit9] Muthukumar R., Karnan M., Elangovan N., Karunanidhi M., Thomas R. (2022). J. Indian Chem. Soc..

[cit10] Ali M. A., Musthafa S. A., Munuswamy-Ramanujam G., Jaisankar V. (2022). Carbohydr. Polym..

[cit11] Zhan M., Jia H., Fan J., Yu H., Amador E., Chen W. (2019). Anal. Chem..

[cit12] Vera S., Vázquez A., Rodriguez R., Del Pozo S., Urruzuno I. a., de Cózar A., Mielgo A., Palomo C. (2021). J. Org. Chem..

[cit13] Collman J. , Hegedus L., Norton J. and Finke R., Priciples and Applications of Organotransition Metal Chemistry, University Science Books, Mill Valley, CA, 1987, p. 324

[cit14] Maharana T. , Nath N., Pradhan H. C., Mantri S., Routaray A. and Sutar A. K., Reactive and Functional Polymers, 2021, p. 105142

[cit15] Luo M., Liu Y., Zhao J., Jiang L., Chen X., Li W., Yang Z., Yan Q., Wang S., Chi Z. (2022). Dyes Pigm..

[cit16] Fernandes C. M., Pina V. G., Alfaro C. G., de Sampaio M. T., Massante F. F., Alvarez L. X., Barrios A. M., Silva J. C. M., Alves O. C., Briganti M. (2022). Colloids Surf., A.

[cit17] Marahel F., Ghaedi M., Montazerozohori M., Biyareh M. N., Kokhdan S. N., Soylak M. (2011). Food Chem. Toxicol..

[cit18] Benmohammed A., Hadji D., Guendouzi A., Mouchaal Y., Djafri A., Khelil A. (2021). J. Electron. Mater..

[cit19] Sun H., Autschbach J. (2013). ChemPhysChem.

[cit20] Mashhadizadeh M. H., Pesteh M., Talakesh M., Sheikhshoaie I., Ardakani M. M., Karimi M. A. (2008). Spectrochim. Acta, Part B.

[cit21] Khalid M., Khan M. U., Shafiq I., Hussain R., Ali A., Imran M., Braga A. A., Fayyaz ur Rehman M., Akram M. S. (2021). R. Soc. Open Sci..

[cit22] Aslam S., Haroon M., Akhtar T., Arshad M., Khalid M., Shafiq Z., Imran M., Ullah A. (2022). ACS Omega.

[cit23] Castet F., Rodriguez V., Pozzo J.-L., Ducasse L., Plaquet A., Champagne B. (2013). Acc. Chem. Res..

[cit24] Khan M. U., Khalid M., Khera R. A., Akhtar M. N., Abbas A., ur Rehman M. F., Braga A. A. C., Alam M. M., Imran M., Wang Y. (2022). Arab. J. Chem..

[cit25] Zhang L., Cai Y., Champagne B., Zhao M. (2017). IET Commun..

[cit26] Champagne B., Bishop D. M. (2003). Adv. Chem. Phys..

[cit27] Ashfaq M., Ali A., Tahir M. N., Khalid M., Assiri M. A., Imran M., Munawar K. S., Habiba U. (2022). Chem. Phys. Lett..

[cit28] Jia J., Wu X., Zhang X., Wang Y., Yang J., Fang Y., Song Y. (2022). Phys. Chem. Chem. Phys..

[cit29] Klikar M., Le Poul P., Ruzicka A., Pytela O., Barsella A., Dorkenoo K. D., Robin-le Guen F., Bureš F., Achelle S. (2017). J. Org. Chem..

[cit30] Autschbach J. (2009). ChemPhysChem.

[cit31] Ali A., Khalid M., Rehman M. F. U., Haq S., Ali A., Tahir M. N., Ashfaq M., Rasool F., Braga A. A. C. (2020). ACS Omega.

[cit32] Pant D., Darla N., Sitha S. (2022). Comput. Theor. Chem..

[cit33] Khalid M., Shafiq I., Mahmood K., Hussain R., ur Rehman M. F., Assiri M. A., Imran M., Akram M. S. (2023). Sci. Rep..

[cit34] Vidyasagar C., Flores B. M., Jiménez-Pérez V., Gurubasavaraj P. (2019). Mater. Today Chem..

[cit35] Hassib H., Razik A. A. (2008). Solid State Commun..

[cit36] Autschbach J. (2007). Coord. Chem. Rev..

[cit37] Lu X. and Champagne B., Pitch analysis-based acoustic echo cancellation over a nonlinear channel, 2002 11th European Signal Processing Conference, IEEE, 2002, pp. 1–4

[cit38] Biswas M., Pilet G., Tercero J., El Fallah M. S., Mitra S. (2009). Inorg. Chim. Acta.

[cit39] Rajaei I., Mirsattari S. N. (2018). J. Mol. Struct..

[cit40] Gharaati A., Abed Y., Mostaghni F. (2022). Bull. Chem. Soc. Ethiop..

[cit41] Arshad M. N., Khalid M., Hani U., Asiri A. M. (2023). J. Phys. Org. Chem..

[cit42] Loukova G., Milov A., Vasiliev V., Minkin V. (2020). Russ. Chem. Bull..

[cit43] Dennington R. , Keith T. and Millam J., Shawnee Mission KS, GaussView, Version, 2009, vol. 5

[cit44] Tenderholt A. L. , PyMOlyze, Version 1.1, Stanford University, 2006, vol. 4, pp. 580–592

[cit45] Sakthivel S., Alagesan T., Muthu S., Abraham C. S., Geetha E. (2018). J. Mol. Struct..

[cit46] Hanwell M. D., Curtis D. E., Lonie D. C., Vandermeersch T., Zurek E., Hutchison G. R. (2012). J. Cheminf..

[cit47] O'boyle N. M., Tenderholt A. L., Langner K. M. (2008). J. Comput. Chem..

[cit48] V. OriginPro, Northamp, MA, USA, 2016

[cit49] Lu T., Chen F. (2012). J. Comput. Chem..

[cit50] Kromann J. C., Steinmann C., Jensen J. H. (2018). J. Chem. Phys..

[cit51] Alparone A. (2013). Chem. Phys..

[cit52] Chattaraj P. K., Roy D. R. (2007). Chem. Rev..

[cit53] Khalid M., Khan M. U., Azhar N., Arshad M. N., Asiri A. M., Braga A. A. C., Akhtar M. N. (2022). Opt. Quantum Electron..

[cit54] Barwiolek M., Jankowska D., Chorobinski M., Kaczmarek-Kędziera A., Łakomska I., Wojtulewski S., Muzioł T. M. (2021). RSC Adv..

[cit55] Ganesan T. S., Elangovan N., Vanmathi V., Sowrirajan S., Chandrasekar S., Murthy K. S., Thomas R. (2022). J. Indian Chem. Soc..

[cit56] Farooq R., Batool Z., Khalid M., Khan M. U., Braga A. A. C., Ragab A. H., Al-Mhyawi S. R., Muhammad G., Shafiq Z. (2022). RSC Adv..

[cit57] Singer K. D., Sohn J. E., King L., Gordon H., Katz H., Dirk C. (1989). J. Opt. Soc. Am. B.

[cit58] Tahir M. N., Khalid M., Islam A., Mashhadi S. M. A., Braga A. A. (2017). J. Mol. Struct..

[cit59] Arshad M. N., Al-Dies A.-A. M., Asiri A. M., Khalid M., Birinji A. S., Al-Amry K. A., Braga A. A. (2017). J. Mol. Struct..

[cit60] Mahmood A., Khan S. U. D., Rana U. A., Janjua M. R. S. A., Tahir M. H., Nazar M. F., Song Y. (2015). J. Phys. Org. Chem..

[cit61] Qiu C. S., Flinn C., Zhao Y. (2019). J. Phys. Org. Chem..

[cit62] Alwadai N., Elqahtani Z. M., Khan S. U. D., Pembere A. M., Badshah A., Mehboob M. Y., Nazar M. F. (2022). J. Phys. Org. Chem..

[cit63] Khan M. U., Mehboob M. Y., Hussain R., Fatima R., Tahir M. S., Khalid M., Braga A. A. C. (2021). J. Phys. Org. Chem..

[cit64] Luise D., d'Alterio M. C., Talarico G., Ciofini I., Labat F. (2022). J. Comput. Chem..

[cit65] Oyeneyin O. E., Ojo N. D., Ipinloju N., James A. C., Agbaffa E. B. (2022). Chem. Afr..

[cit66] Reed A. E., Curtiss L. A., Weinhold F. (1988). Chem. Rev..

[cit67] Foster a. J., Weinhold F. (1980). J. Am. Chem. Soc..

[cit68] Mahmood A., Irfan A., Ahmad F., Janjua M. R. S. A. (2021). Comput. Theor. Chem..

[cit69] Mahmood A., Khan S. U.-D., Rana U. A., Tahir M. H. (2019). Arab. J. Chem..

[cit70] Kosower E. M. (1958). J. Am. Chem. Soc..

[cit71] Sun Y.-X., Hao Q.-L., Wei W.-X., Yu Z.-X., Lu L.-D., Wang X., Wang Y.-S. (2009). J. Mol. Struct.: THEOCHEM.

[cit72] Mehboob M. Y., Hussain R., Jamil S., Ahmed M., Khan M. U., Haroon M., Janjua M. R. S. A. (2022). J. Phys. Org. Chem..

