# Supplementary material for: Synthesis, characterization and NLO properties of 1,4-phenylenediamine-based Schiff bases: a combined theoretical and experimental approach
Source: RSC Adv. 2024 Jan 30;14(6):4221–9. doi: 10.1039/d3ra07642c (PMC10826287; doi:10.1039/d3ra07642c)
Supplement: RA-014-D3RA07642C-s001 [file RA-014-D3RA07642C-s001.pdf]

Electronic Supplementary Material (ESI) for RSC Advances.
This journal is © The Royal Society of Chemistry 2024

Synthesis, Characterization and NLO Properties of 1,4-phenylenediamine Based Schiff's Bases: A Combined Theoretical and Experimental Approach

Muhammad Tahir,[a] Hina Aftab,[a] Iqra Shafiq,[b,c] Muhammad Khalid, [*b,c] Saadia Haq,[b,c] Attalla F. El-kott,[d,e] Mohamed Abdellatif Zein[f,g], Umme-Hani[b,c] and Zahid Shafiq[*a]

[a]*Institute of Chemical Sciences, Bahauddin Zakariya University, Multan 60800, Pakistan*

[b]*Institute of Chemistry, Khwaja Fareed University of Engineering & Information Technology, Rahim Yar Khan, 64200, Pakistan*

[c]*Centre for Theoretical and Computational Research, Khwaja Fareed University of Engineering & Information Technology, Rahim Yar Khan, 64200, Pakistan*

[d] *Biology Department, College of Science, King Khalid University, Abha, Saudi Arabia.*

[e] *Zoology Department, College of Science, Damanhour University, Egypt*

[f] *Chemistry Department, University College of AlWajh, University of Tabuk, Tabuk, Saudi Arabia*

[g] *Chemistry Department, Faculty of Science, Damanhour University, Egypt*

Corresponding Authors

Prof. Dr. Zahid Shafiq: zahidshafiq@bzu.edu.pk
Dr. Muhammad Khalid: khalid@iq.usp.br; muhammad.khalid@kfueit.edu.pk

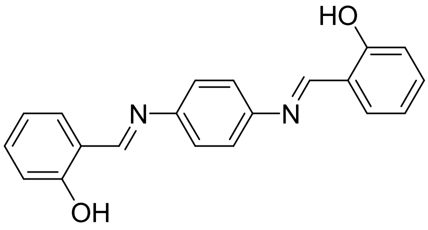

(3a)

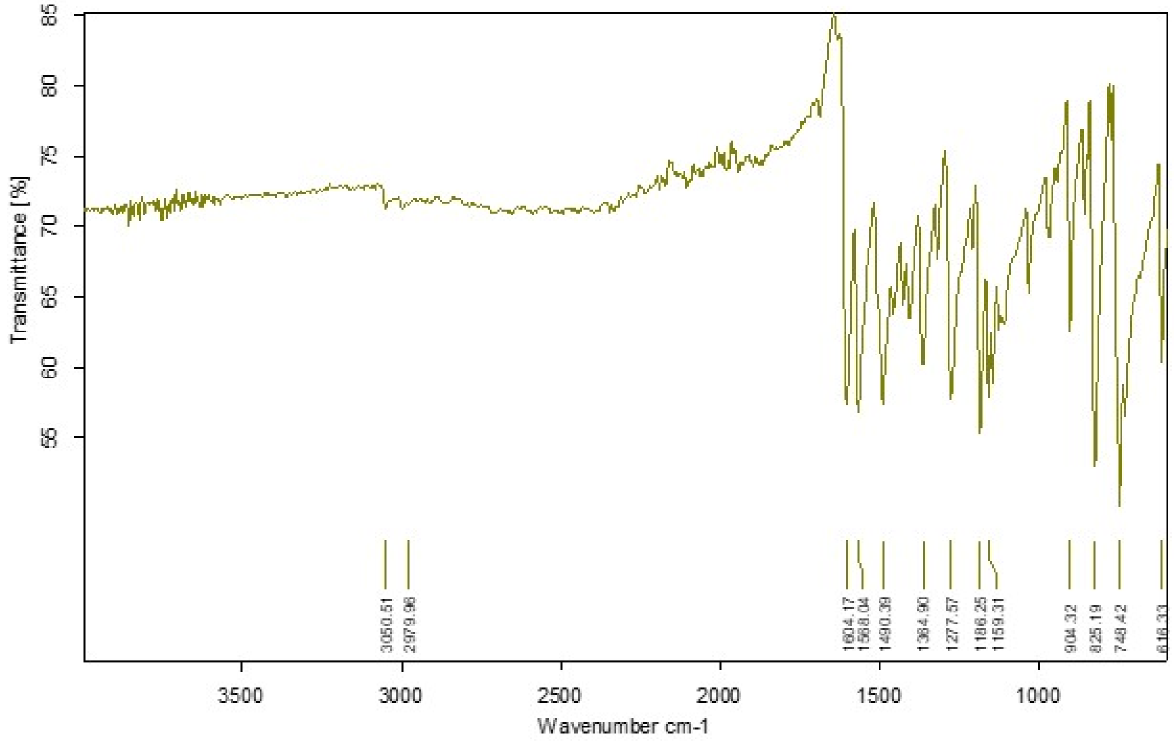

Figure S1: FT- IR spectrum of **3a** compound

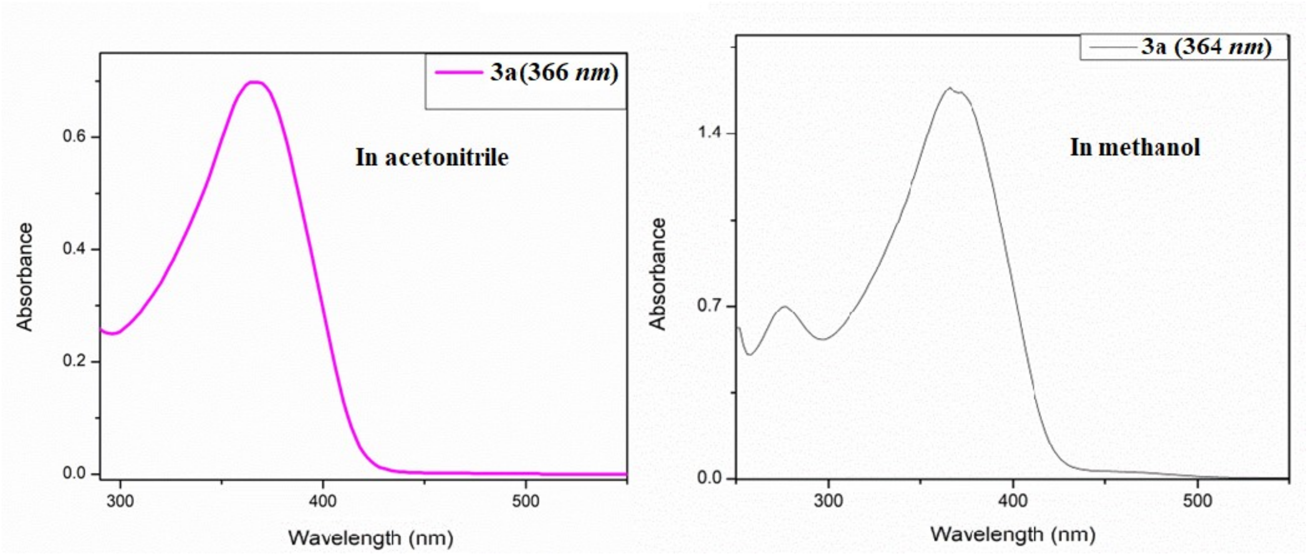

Figure S2: UV-Vis spectra of **3a** in various media

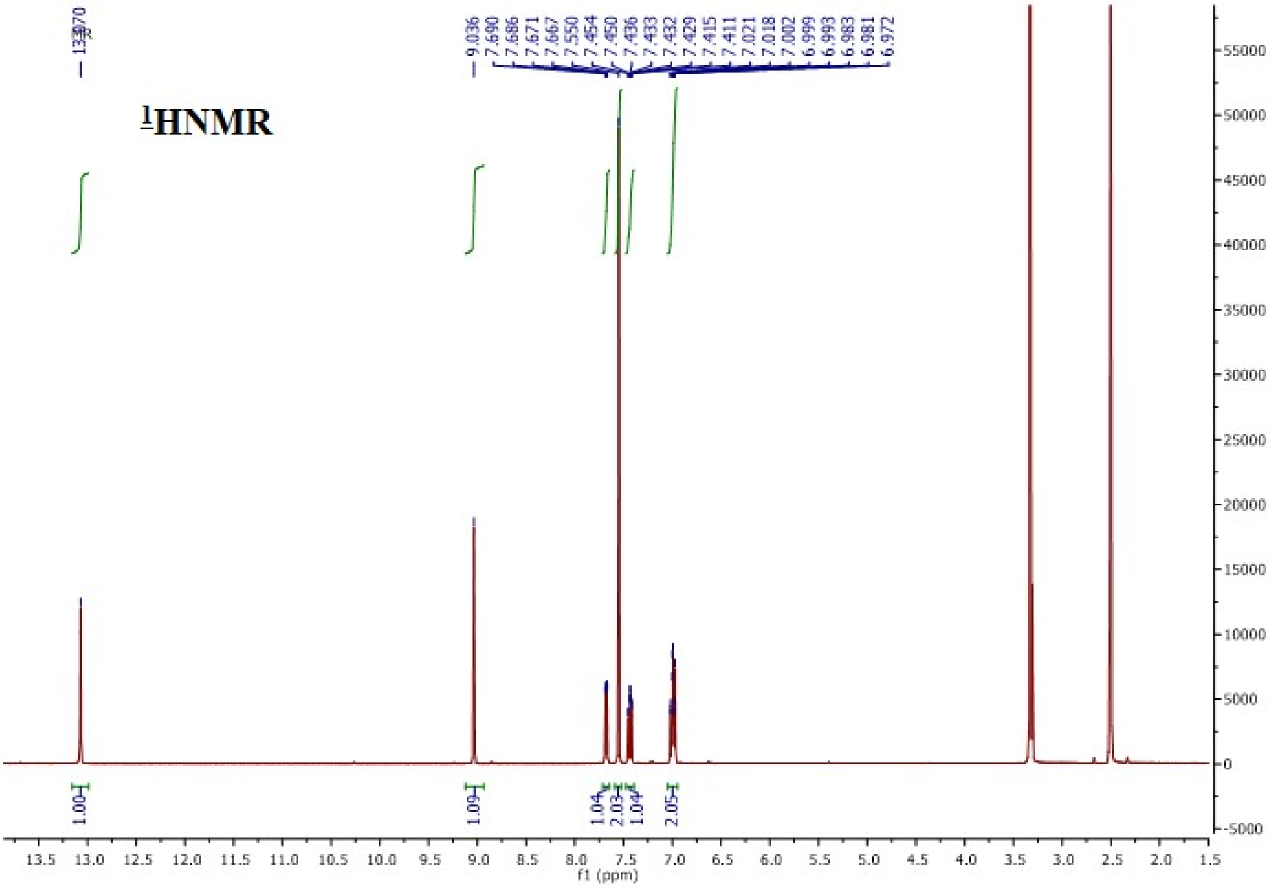

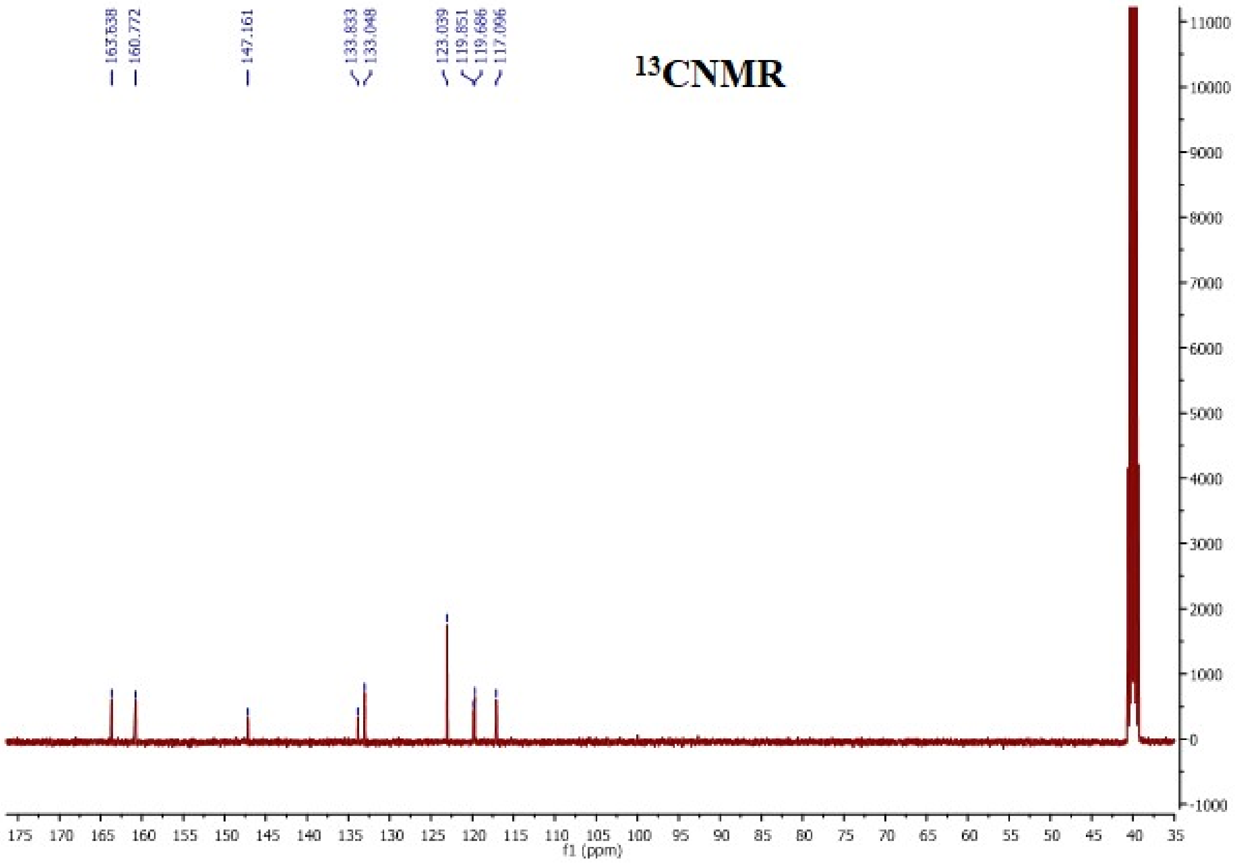

Figure S3: ^{1}HNMR and ^{13}CNMR spectral representation of **3a** compound

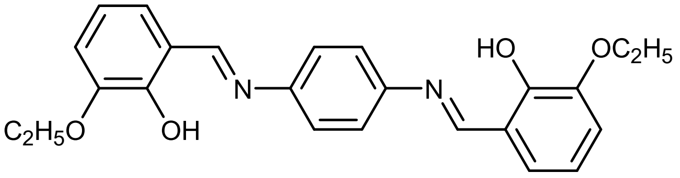

(3b)

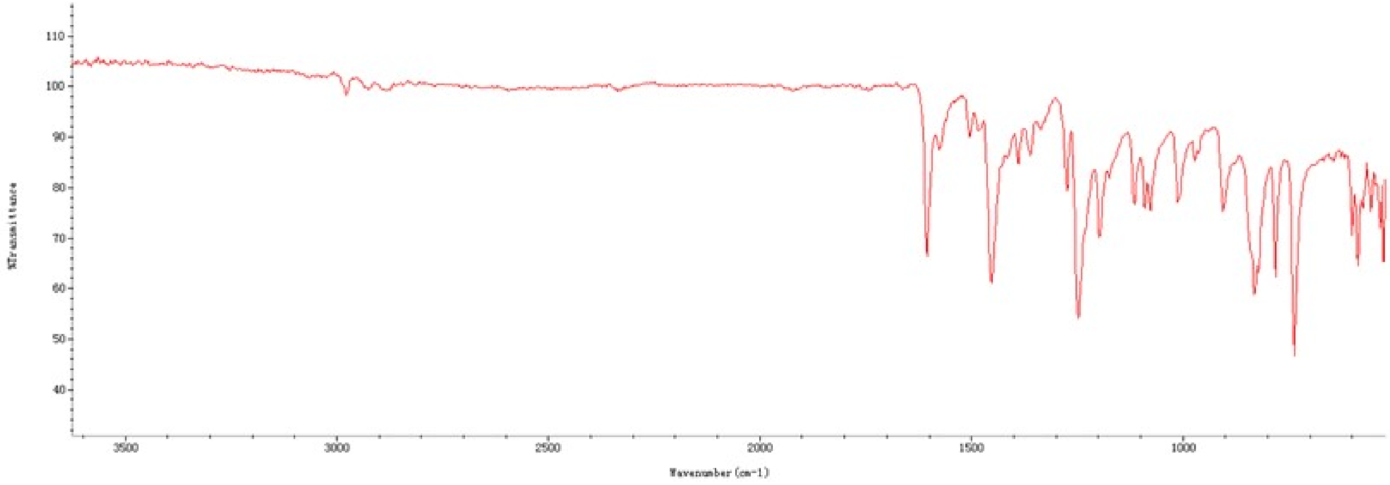

Figure S4: FT- IR spectrum of **3b** compound

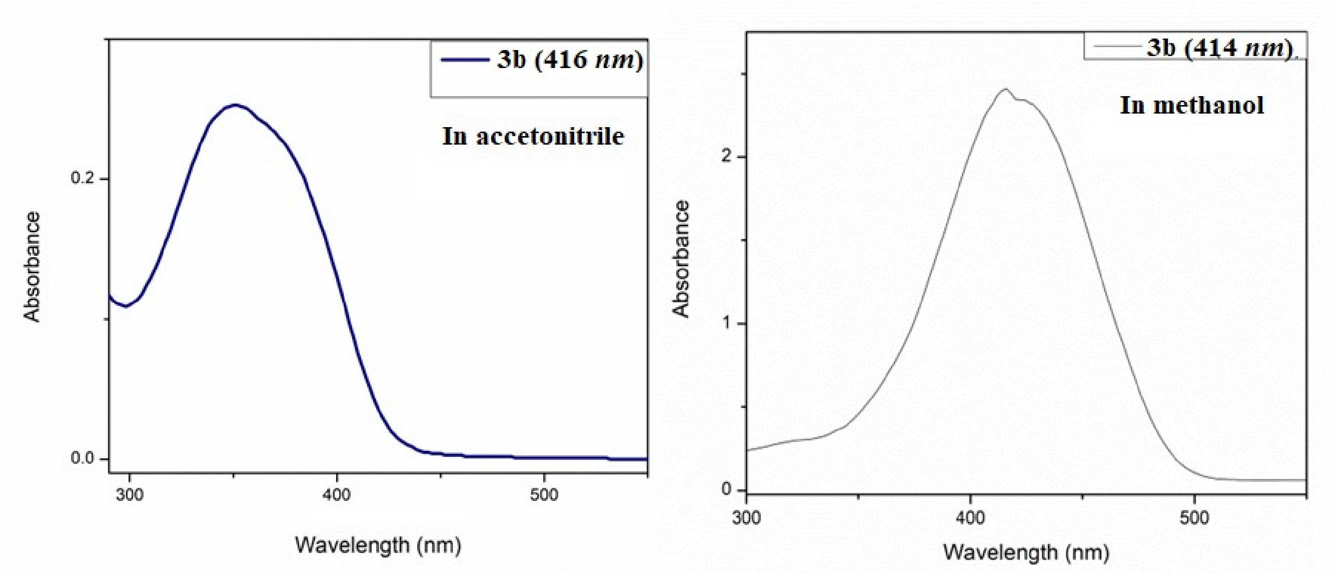

Figure S5: UV-Vis spectra of **3b** in various media

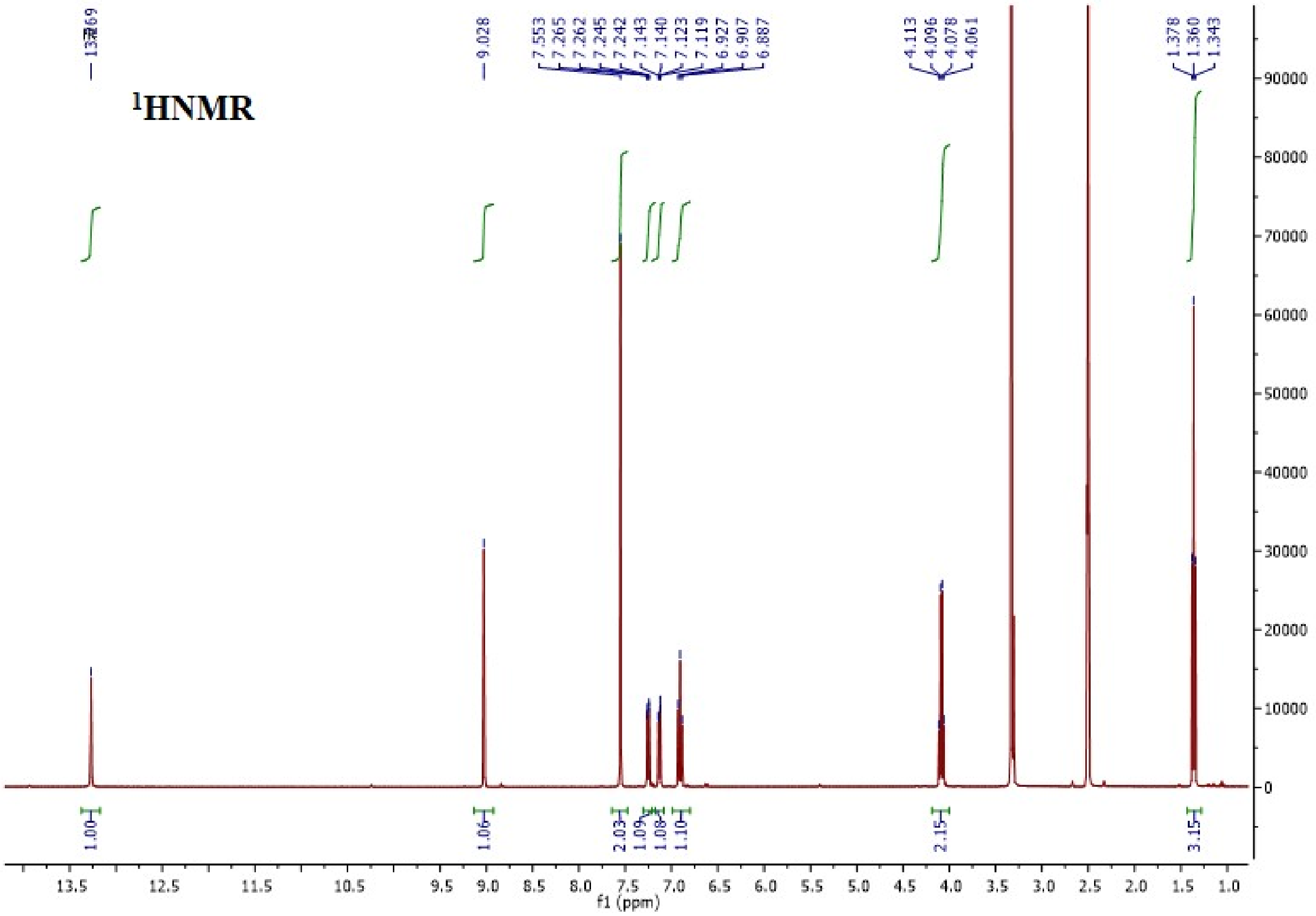

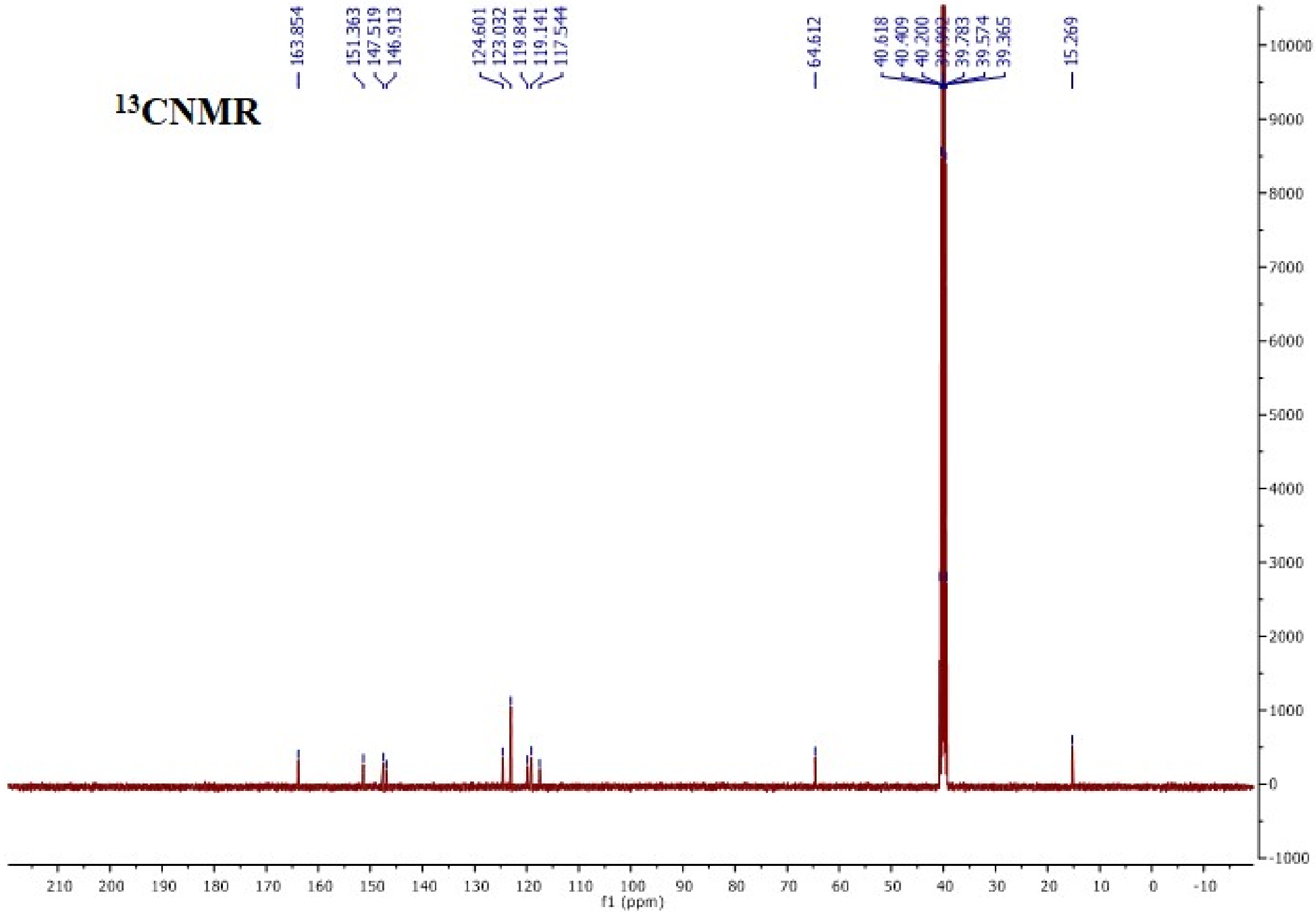

Figure S6: ^{1}HNMR and ^{13}CNMR spectral representation of **3b** compound

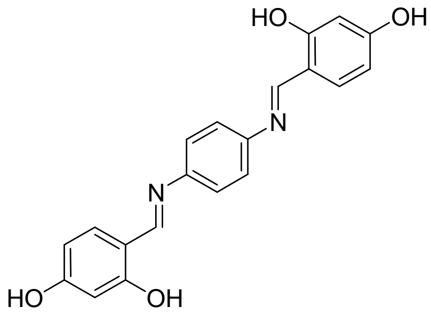

(3c)

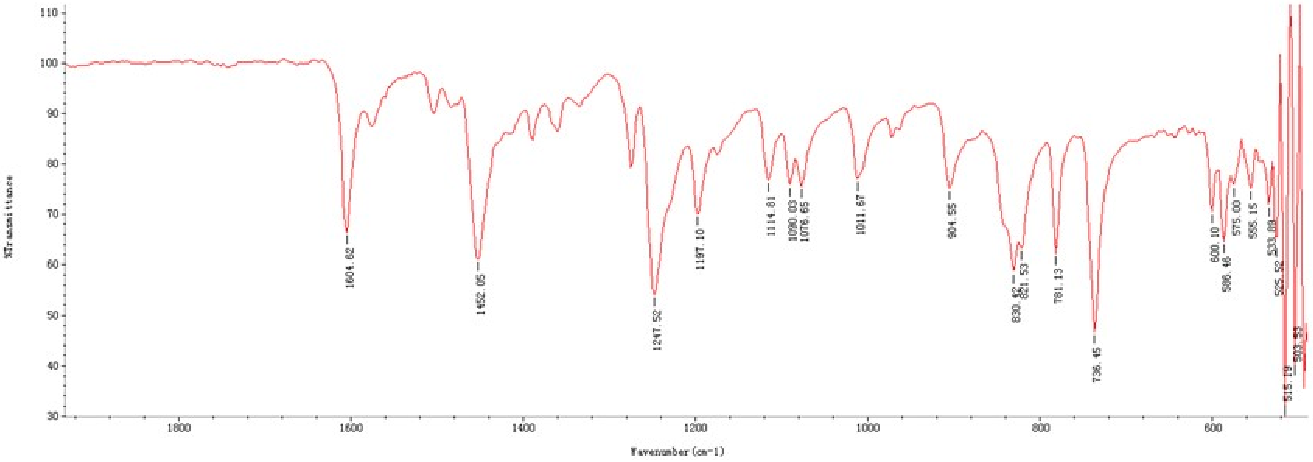

Figure S7: FT- IR spectral representation of **3c** compound

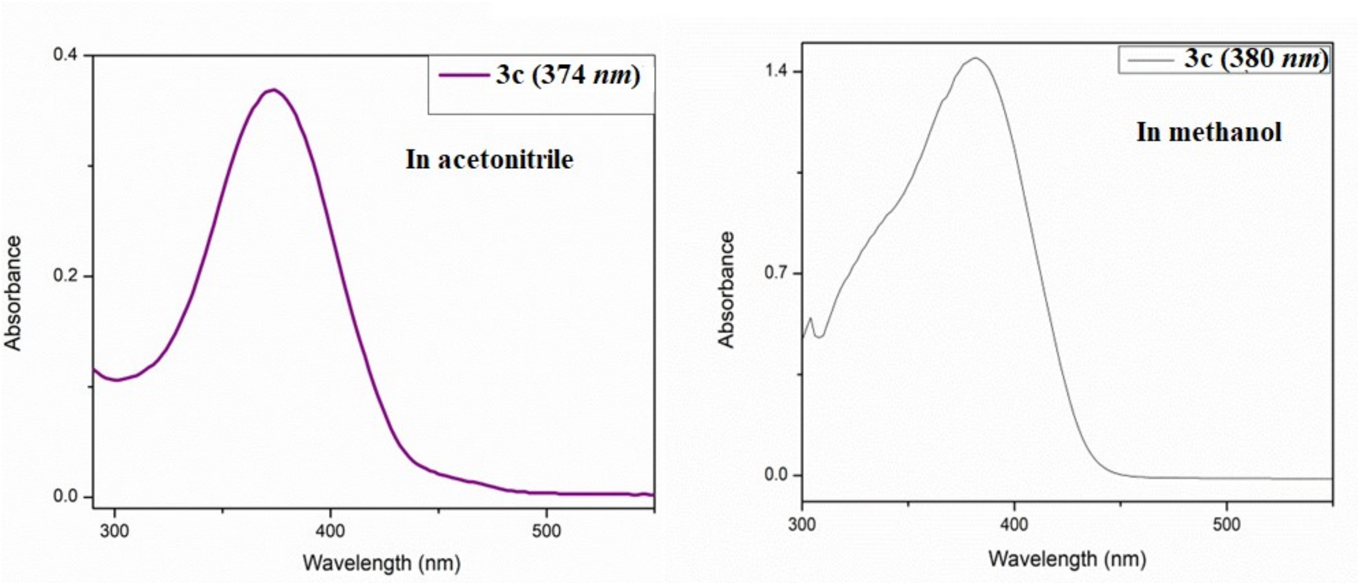

Figure S8: UV-Vis spectral representation of **3c** in various media

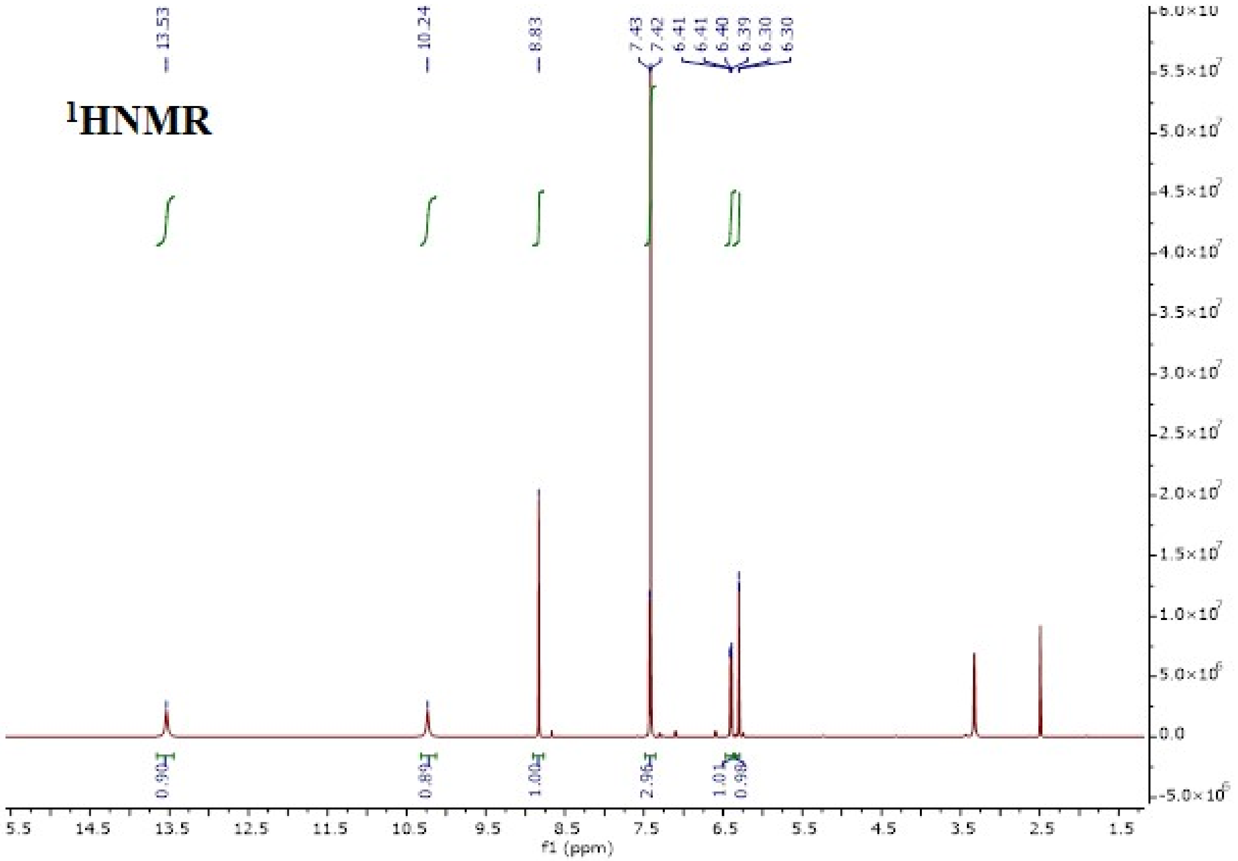

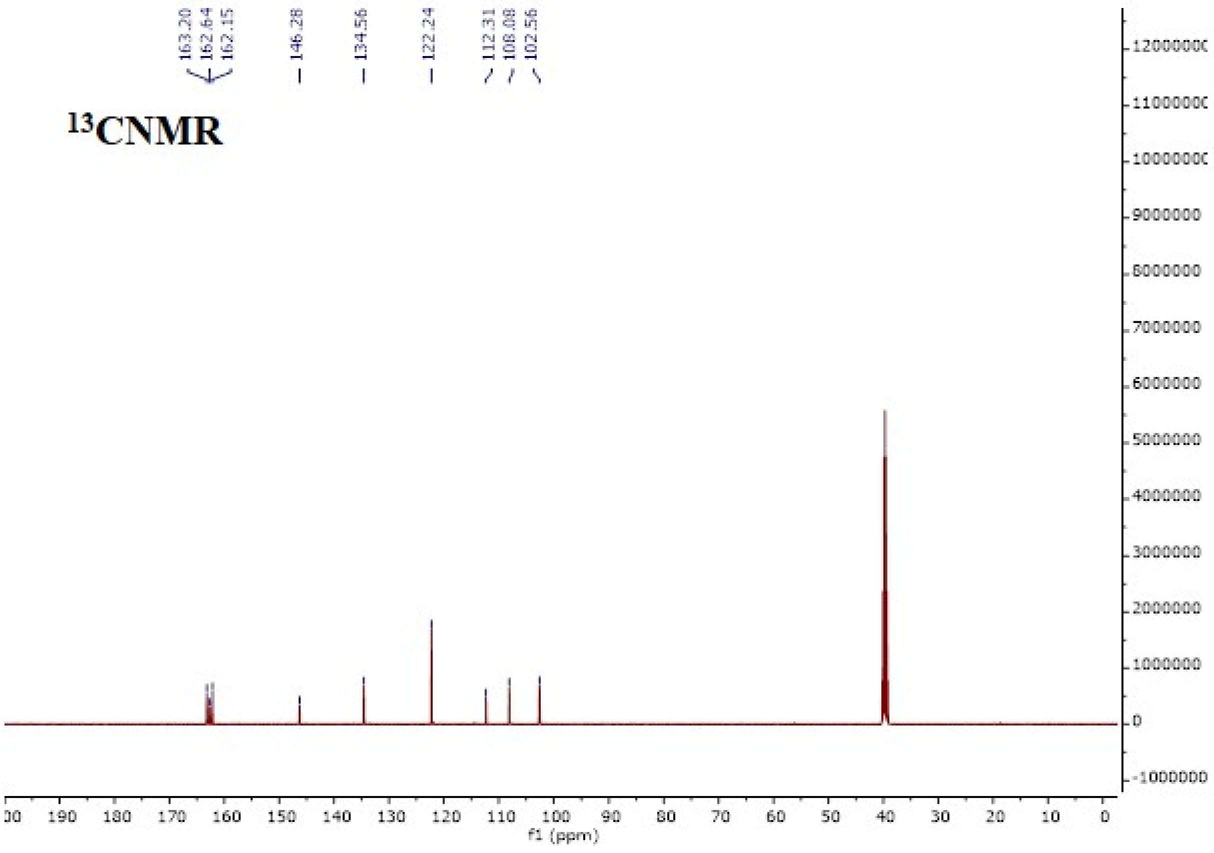

Figure S9: ^{1}HNMR and ^{13}CNMR spectral representation of **3c** compound

3a

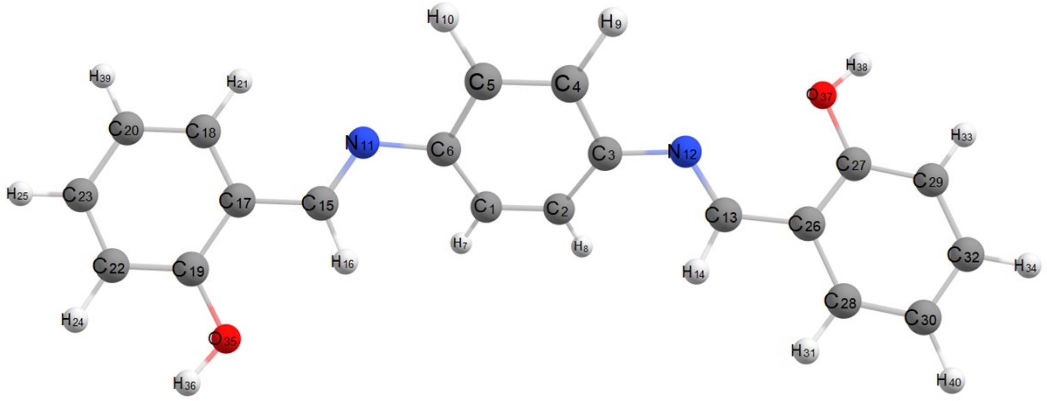

3b

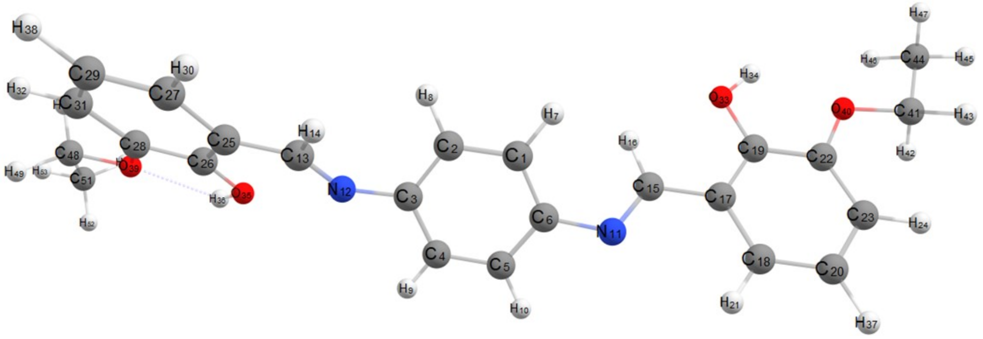

3c

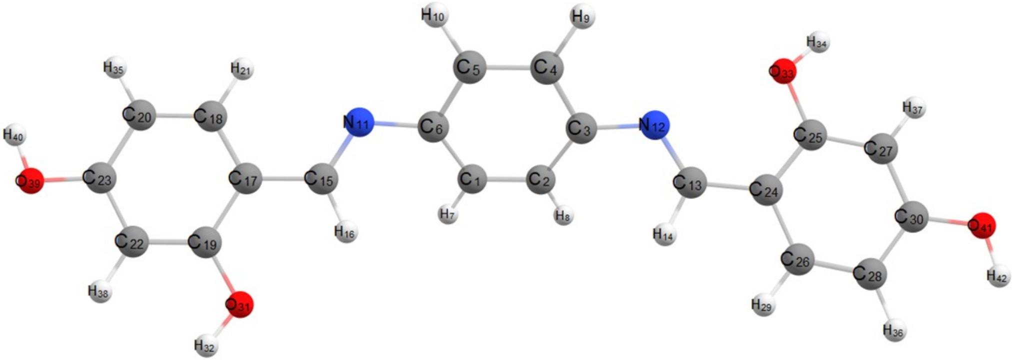

Figure S10: Optimized structures of studied compounds.

Table S1: Calculated energies (*E*) and energy gap (ΔE) of HOMO-1, LUMO+1, HOMO-2 and LUMO+2 for **3a-3c.**

Compound	HOMO-1	LUMO+1	ΔE	HOMO-2	LUMO+2	ΔE
**3a**	-6.817	-1.399	5.418	-6.957	-0.334	6.623
**3b**	-6.425	-1.340	5.085	-6.475	-0.326	6.149
**3c**	-6.624	-1.161	5.463	-6.955	-0.278	6.677

Table S2: Natural bond orbital (NBO) analysis of **3a.**

Donor(*i*)	Type	Acceptor(*j*)	Type	*E*(2) [*kcal/mol*]	*E*(*j*)-*E*(*i*) [*a.u*]	*F*(*i,j*) [*a.u*]
C29-C32	π	C26-C27	π*	26.25	0.29	0.081
C28-C30	π	C29-C32	π*	25.89	0.3	0.079
C22-C23	π	C17-C19	π*	25.87	0.29	0.08
C18-C20	π	C22-C23	π*	25.85	0.29	0.078
C26-C27	π	C28-C30	π*	24.86	0.3	0.079
C17-C19	π	C18-C20	π*	24.11	0.31	0.079
C2-C3	π	C1-C6	π*	22.68	0.3	0.074

C18-C20	π	C17-C19	π*	17.73	0.29	0.066
N12-C13	π	C2-C3	π*	10.62	0.38	0.062
C1-C6	π	N11-C15	π*	8.61	0.3	0.048
C2-C3	π	N12-C13	π*	8.22	0.3	0.047
N11-C15	π	C17-C19	π*	7.57	0.37	0.052
N12-C13	π	C26-C27	π*	7.09	0.37	0.05
C17-C19	π	C17-C19	π*	1.97	0.29	0.022
C26-C27	π	C26-C27	π*	1.95	0.29	0.021
C19-C22	σ	C17-C19	σ*	5.29	1.29	0.074
O37-H38	σ	C26-C27	σ*	5.21	1.31	0.074
C27-C29	σ	C26-C27	σ*	5.15	1.28	0.073
C18-H21	σ	C17-C19	σ*	4.95	1.08	0.065
O35-H36	σ	C17-C19	σ*	4.82	1.32	0.072
C28-H31	σ	C26-C27	σ*	4.79	1.08	0.064
C13-H14	σ	C26-C27	σ*	4.76	1.09	0.065
C1-C6	σ	C5-C6	σ*	4.7	1.29	0.069
C2-C3	σ	C3-C4	σ*	4.68	1.29	0.069
C15-C17	σ	C6-N11	σ*	4.65	1.14	0.065
C27-C29	σ	C32-H34	σ*	2.15	1.15	0.044
C23-H25	σ	C20-C23	σ*	0.64	1.11	0.024
N11-C15	σ	C15-H16	σ*	0.62	1.26	0.025
C15-H16	σ	N11-C15	σ*	0.6	1.15	0.024
C22-H24	σ	C19-C22	σ*	0.6	1.1	0.023
C27-C29	σ	O37-H38	σ*	0.57	1.14	0.023
C29-H33	σ	C27-C29	σ*	0.57	1.09	0.022
N12-C13	σ	C13-H14	σ*	0.56	1.26	0.024
C19-C22	σ	C19-O35	σ*	0.56	1.09	0.022
C27-C29	σ	C27-O37	σ*	0.54	1.1	0.022
C19-C22	σ	O35-H36	σ*	0.52	1.14	0.022
N12	LP(1)	C13-H14	σ*	13.09	0.72	0.088
N11	LP(1)	C15-H16	σ*	12.45	0.73	0.087
N11	LP(1)	C1-C6	σ*	6.97	0.93	0.073
O35	LP(1)	C19-C22	σ*	6.35	1.19	0.078
N12	LP(1)	C13-C26	σ*	2.5	0.85	0.042
N11	LP(1)	C5-C6	σ*	0.81	0.94	0.025
N12	LP(1)	C3-C4	σ*	0.71	0.93	0.024
O37	LP(2)	C26-C27	π*	33.73	0.37	0.107
O35	LP(2)	C17-C19	π*	31.62	0.37	0.105
N12	LP(1)	C2-C3	π*	5.56	0.39	0.045
N11	LP(1)	C1-C6	π*	4.61	0.39	0.041

Table S3: Natural bond orbital (NBO) analysis of **3b.**

**Donor(*i*)**	**Type**	**Acceptor(*j*)**	**Type**	***E*(2) [*kcal/mol*]**	***E*(*j*)-*E*(*i*) [*a.u*]**	***F*(*i,j*) [*a.u*]**
C2-C3	π	C4-C51	π*	9.85	0.3	0.07
C25-C26	π	C28-C31	π*	9.6	0.3	0.068
C17-C19	π	N11-C15	π*	9.38	0.31	0.073
C17-C19	π	C22-C23	π*	9.33	0.3	0.068
C28-C31	π	C27-C29	π*	9.04	0.32	0.07
C22-C23	π	C18-C20	π*	8.83	0.32	0.07

C1-C6	π	N11-C15	π*	8.45	0.3	0.047
N11-C15	π	C1-C61	π*	0.76	0.38	0.062
C27-C29	π	C28-C31	π*	0.66	0.29	0.071
C28-C31	π	C25-C26	π*	0.61	0.31	0.073
C18-C20	π	C22-C23	π*	0.46	0.29	0.071
C22-C23	π	C17-C19	π*	0.39	0.31	0.073
N12-C13	π	C2-C31	π*	0.36	0.38	0.061
C1-C6	π	C4-C52	π*	0.02	0.3	0.071
C26-C28	σ	C13-C25	σ*	3.49	1.22	0.058
C26-C28	σ	C25-C26	σ*	5.04	1.28	0.072
C26-C28	σ	C26-O35	σ*	0.5	1.09	0.021
C26-C28	σ	C28-C31	σ*	5.1	1.3	0.073
C26-C28	σ	C28-O39	σ*	0.81	1.09	0.026
C26-C28	σ	C31-H32	σ*	2.31	1.13	0.046
C26-C28	σ	O35-H36	σ*	0.62	1.14	0.024
C26-C28	σ	O39-C48	σ*	3.89	1.01	0.056
C26-O35	σ	C25-C26	σ*	1.86	1.52	0.048
C26-O35	σ	C25-C27	σ*	1.54	1.52	0.043
N11-C15	σ	N11-C15	σ*	0.69	0.58	0.062
N12-C13	σ	N12-C13	σ*	0.77	0.58	0.064
O40	LP(2)	C22-C23	π*	9.82	0.37	0.1
O39	LP(2)	C28-C31	π*	9.35	0.37	0.099
N12	LP(1)	C2-C3	π*	5.92	0.39	0.046
N11	LP(1)	C1-C6	π*	5.13	0.39	0.043
O35	LP(2)	C25-C26	π*	4	0.37	0.107
O33	LP(2)	C17-C19	π*	1.99	0.37	0.105
O40	LP(1)	C22-C23	σ*	7.19	1.16	0.082
O39	LP(1)	C28-C31	σ*	7.06	1.16	0.081
N11	LP(1)	C1-C6	σ*	6.77	0.94	0.072
O40	LP(1)	C41-H43	σ*	0.63	0.95	0.022
O40	LP(1)	C41-H42	σ*	0.62	0.95	0.022
O39	LP(1)	C48-H49	σ*	0.61	0.95	0.022

Table S4: Natural bond orbital (NBO) analysis of **3c.**

**Donor(*i*)**	**Type**	**Acceptor(*j*)**	**Type**	***E*(2) [*kcal/mol*]**	***E*(*j*)-*E*(*i*) [*a.u*]**	***F*(*i,j*) [*a.u*]**
C1-C6	π	C2-C3	π*	22.34	0.3	0.073
C1-C6	π	C4-C5	π*	20.06	0.3	0.071
C1-C6	π	N11-C15	π*	8.49	0.3	0.047
C2-C3	π	C1-C6	π*	22.62	0.3	0.074
C2-C3	π	C4-C5	π*	19.9	0.3	0.07
C2-C3	π	N12-C13	π*	8.2	0.3	0.047
C4-C5	π	C1-C6	π*	20.75	0.3	0.072
C17-C19	π	C18-C20	π*	27.76	0.3	0.083
C24-C26	π	C28-C30	π*	15.4	0.28	0.06
C25-C27	π	C24-C26	π*	13.61	0.3	0.059
C25-C27	π	C25-C27	π*	1.88	0.3	0.022
C25-C27	π	C28-C30	π*	29.06	0.3	0.085
C28-C30	π	C24-C26	π*	31.78	0.3	0.088
C28-C30	π	C25-C27	π*	14.99	0.29	0.059

C28-C30	π	C28-C30	π*	2.18	0.29	0.023
C1-C2	σ	C1-C6	σ*	3.97	1.29	0.064
C1-C2	σ	C1-H7	σ*	1.34	1.13	0.035
C1-C2	σ	C2-C3	σ*	3.97	1.29	0.064
C1-C2	σ	C2-H8	σ*	1.33	1.13	0.035
C1-C2	σ	C3-N12	σ*	4.06	1.17	0.062
C1-C2	σ	C6-N11	σ*	4.11	1.17	0.062
C1-C6	σ	C1-C2	σ*	3.94	1.31	0.064
C1-C6	σ	C1-H7	σ*	1.22	1.12	0.033
C1-C6	σ	C2-H8	σ*	2.35	1.13	0.046
C1-C6	σ	C5-C6	σ*	4.67	1.29	0.069
C28-H36	σ	C28-C30	σ*	0.62	1.09	0.023
C28-H36	σ	C30-O41	σ*	1.03	0.9	0.027
C30-O41	σ	C25-C27	σ*	1.51	1.54	0.043
C30-O41	σ	C26-C28	σ*	1.09	1.57	0.037
C30-O41	σ	C27-C30	σ*	1.08	1.54	0.037
C30-O41	σ	C28-C30	σ*	1.21	1.53	0.039
O31-H32	σ	C17-C19	σ*	4.78	1.32	0.071
O33-H34	σ	C24-C25	σ*	5.16	1.31	0.074
O39-H40	σ	C22-C23	σ*	4.79	1.33	0.072
O41-H42	σ	C27-C30	σ*	4.81	1.33	0.072
N11	LP(1)	C1-C6	σ*	6.99	0.93	0.073
N11	LP(1)	C5-C6	σ*	0.79	0.93	0.025
O33	LP(1)	C25-C27	σ*	6.2	1.2	0.077
O39	LP(1)	C20-C23	σ*	6.54	1.19	0.079
O41	LP(1)	C28-C30	σ*	6.59	1.19	0.079
N11	LP(1)	C1-C6	π*	4.65	0.39	0.041
N12	LP(1)	C2-C3	π*	5.52	0.39	0.044
O31	LP(2)	C17-C19	π*	32.5	0.37	0.107
O33	LP(2)	C25-C27	π*	35.86	0.37	0.109
O39	LP(2)	C22-C23	π*	33.92	0.37	0.107
O41	LP(2)	C28-C30	π*	33.75	0.37	0.107

Table S5: Wavelength, excitation energy and oscillator strength of investigated compound **3a** at **M06/6-311G(d,p)** in solvent phase.

NO	DFT λ (*nm*)	*E*(*eV*)	f_{os}	MO contributions
1	384.757	3.222	1.104	H→L (94%),
2	326.652	3.796	0.010	H→L+1 (79%), H-6→L (8%), H-5→L+1 (3%), H-4→L (4%)
3	306.497	4.045	0.072	H-2→L (35%), H-1→L (46%), H-2→L+1 (8%), H-1→L+1 (2%)
4	302.305	4.101	0.119	H-2→L (38%), H-1→L (35%), H-1→L+1 (10%), H-6→L+1 (2%), H-2→L+1 (5%)
5	282.469	4.389	0.006	H-5→L (10%), H-4→L (46%), H→L+2 (19%), H-6→L (6%), H→L+1 (9%)
6	278.779	4.447	0.262	H-5→L (25%), H-4→L (12%), H-3→L (17%), H-7→L (5%), H-6→L (7%), H-6→L+1 (6%), H-4→L+1 (3%), H-3→L+1 (5%), H-2→L (3%), H-1→L+1 (3%), H→L+2 (5%)

MO=molecular orbital, H=HOMO, L=LUMO, f_{os}= oscillator strength, wavelength= λ (*nm*)

Table S6: Wavelength, excitation energy and oscillator strength of investigated compound **3b** at **M06/6-311G(d,p)** in solvent phase.

NO	DFT λ (*nm*)	$E(eV)$	f_{os}	MO contributions
1	378.635	3.275	1.234	H→L (93%)
2	330.722	3.749	0.047	H-2→L (29%), H-1→L (52%), H-2→L+1 (9%), H→L+1 (7%)
3	327.256	3.789	0.041	H-2→L (53%), H-1→L (27%), H-1→L+1 (13%),
4	323.204	3.836	0.002	H→L+1 (68%), H-6→L (8%), H-5→L (4%), H-4→L+1 (5%), H-2→L+1 (5%), H-1→L (4%)
5	284.648	4.356	0.436	H-6→L+1 (10%), H-4→L (52%), H-7→L (9%), H-5→L (5%), H-5→L+1 (4%), H-3→L (7%), H-3→L+1 (5%)
6	282.000	4.397	0.026	H-5→L (42%), H→L+1 (16%), H→L+2 (11%), H-6→L (6%), H-4→L (7%), H-4→L+1 (3%), H-2→L+1 (4%), H-1→L (3%), H-1→L+1 (2%)

MO=molecular orbital, H=HOMO, L=LUMO, f_{os}= oscillator strength, wavelength= λ (*nm*)

Table S7: Wavelength, excitation energy and oscillator strength of investigated compound **3c** at **M06/6-311G(d,p)** in solvent phase.

NO	DFT λ (*nm*)	$E(eV)$	f_{os}	MO contributions
1	382.172	3.244	1.332	H→L (94%),
2	319.926	3.875	0.012	H→L+1 (80%), H-6→L (7%), H-5→L (3%), H-2→L+1 (4%)
3	298.664	4.151	0.090	H-2→L (21%), H-1→L (59%), H-3→L (3%), H-2→L+1 (4%)
4	292.913	4.233	0.230	H-2→L (50%), H-1→L (23%), H-7→L (4%), H-6→L+1 (4%), H-2→L+1 (3%), H-1→L+1 (5%)
5	279.988	4.428	0.006	H-5→L (43%), H→L+2 (44%), H→L+1 (4%)
6	276.417	4.485	0.100	H-4→L (55%), H-3→L+1 (11%), H-7→L (3%), H-3→L (6%), H-1→L+1 (3%), H-1→L+4 (3%), H→L+3 (4%), H→L+4 (5%)

MO=molecular orbital, H=HOMO, L=LUMO, f_{os}= oscillator strength, wavelength= λ (*nm*)

Table S8: Dipole polarizability and major contributing tensor (*e.s.u.*) of **3a-3c**

Dipole moment	**3a**	**3b**	**3c**
μ_x	0.3510	-0.1877	0.0997
μ_y	4.4568	4.2557	4.7915
μ_z	0.0940	0.1085	0.0158
μ_{total}	4.4716	4.2612	4.7926
Polarizability			
α_{xx}	1.02×10^{-22}	1.15×10^{-22}	1.10×10^{-22}
α_{yy}	5.12×10^{-23}	5.30×10^{-23}	5.17×10^{-23}
α_{zz}	2.16×10^{-23}	4.10×10^{-23}	2.24×10^{-23}
α_{total}	5.83×10^{-23}	6.99×10^{-23}	6.16×10^{-23}

2nd Hyper pol.			
γ_X	7.16×10^{-34}	7.42×10^{-34}	8.13×10^{-34}
γ_Y	1.11×10^{-35}	1.79×10^{-35}	1.08×10^{-35}
γ_Z	9.17×10^{-37}	8.22×10^{-36}	1.00×10^{-36}
Average<γ>	7.28×10^{-34}	7.68×10^{-34}	8.24×10^{-34}
Magnitude of γ	7.16×10^{-34}	7.42×10^{-34}	8.13×10^{-34}

Table S9: Frequency dependent Second hyperpolarizability (*e.s.u.*) of **3a-3c**

**Parameters**	**Frequency ω**	**3a**	**3b**	**3c**
γ(−ω,ω,0,0)	0.000	7.28×10^{-34}	7.68×10^{-34}	8.24×10^{-34}
	1907.21*nm*	6.26×10^{-34}	6.80×10^{-34}	7.12×10^{-34}
γ(−2ω,ω,ω,0)	0.000	7.28×10^{-34}	7.68×10^{-34}	8.24×10^{-34}
	1907.21*nm*	6.80×10^{-34}	7.45×10^{-34}	7.76×10^{-34}

Table S10: The computed first hyperpolarizability (β_{tot}) and major contributing tensors (*e.s.u.*) of **3a-3c**

**Polarizability**	**3a**	**3b**	**3c**
$\boldsymbol{\beta_{xxx}}$	8.48×10^{-30}	8.57×10^{-30}	3.76×10^{-30}
$\boldsymbol{\beta_{xxy}}$	-9.09×10^{-30}	-3.28×10^{-30}	-8.23×10^{-31}
$\boldsymbol{\beta_{xyy}}$	-1.00×10^{-30}	-2.96×10^{-30}	6.33×10^{-31}
$\boldsymbol{\beta_{yyy}}$	-1.58×10^{-30}	3.03×10^{-30}	-3.07×10^{-30}
$\boldsymbol{\beta_{xxz}}$	4.26×10^{-30}	4.77×10^{-30}	4.42×10^{-30}
$\boldsymbol{\beta_{yyz}}$	2.12×10^{-31}	1.93×10^{-30}	1.11×10^{-30}
$\boldsymbol{\beta_{xzz}}$	-6.47×10^{-32}	1.22×10^{-30}	-8.41×10^{-32}
$\boldsymbol{\beta_{yzz}}$	-1.09×10^{-31}	-1.33×10^{-30}	-5.51×10^{-32}
$\boldsymbol{\beta_{zzz}}$	-8.59×10^{-32}	3.49×10^{-30}	-8.38×10^{-32}
$\boldsymbol{\beta_{total}}$	1.40×10^{-30}	1.23×10^{-30}	8.00×10^{-30}

Table S11: Frequency dependent First hyperpolarizability (*e.s.u.*) of **3a-3c**

	**Parameters**	**Frequency ω**	**3a**	**3b**	**3c**
Static	β (−ω;ω,0,)	0.000	1.40×10^{-29}	1.23×10^{-29}	8.00×10^{-30}
	β (-2, ω;ω,ω)	0.000	1.40×10^{-29}	1.23×10^{-29}	8.00×10^{-30}
Specific	β (−ω;ω,0)	1907.21nm	5.05×10^{-23}	6.14×10^{-23}	5.36×10^{-23}
	β (−2ω;ω,ω)	1907.21nm	5.05×10^{-23}	6.14×10^{-23}	5.36×10^{-23}

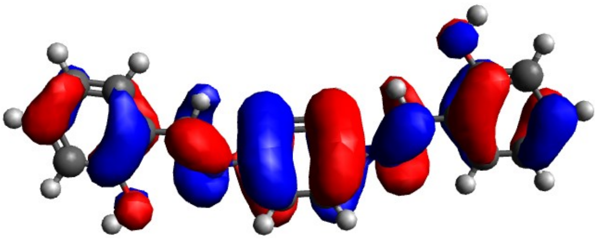

HOMO

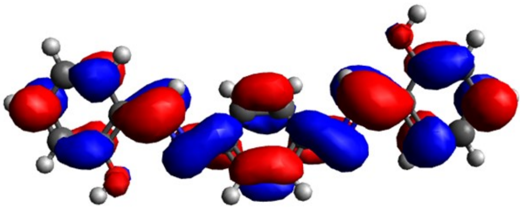

LUMO

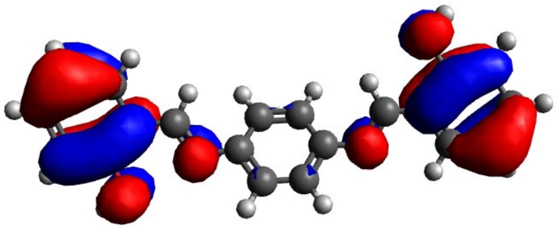

HOMO -1

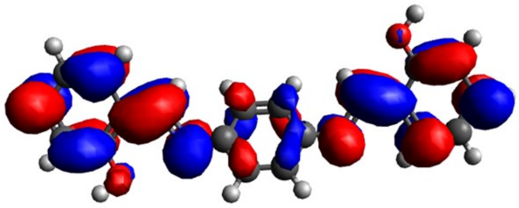

LUMO +1

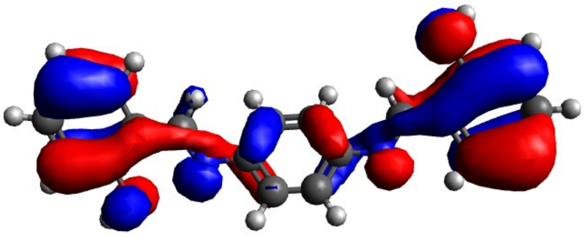

HOMO -2

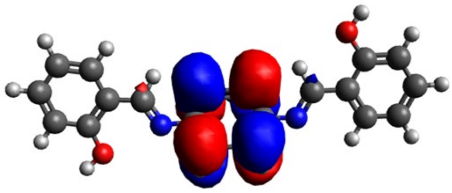

LUMO +2

(3a)

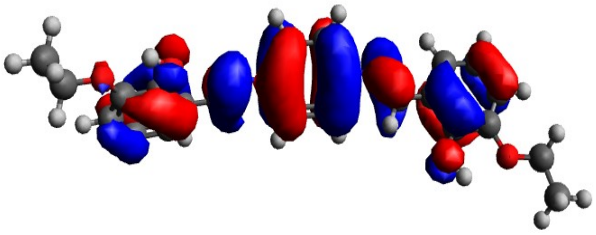

HOMO

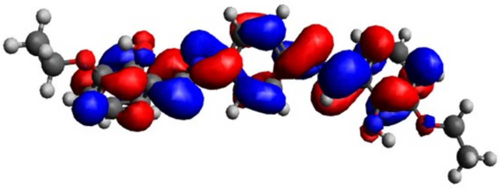

LUMO

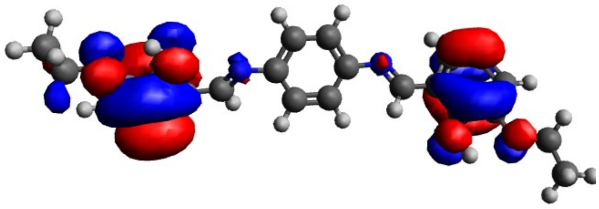

HOMO -1

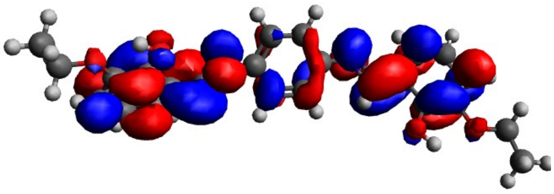

LUMO +1

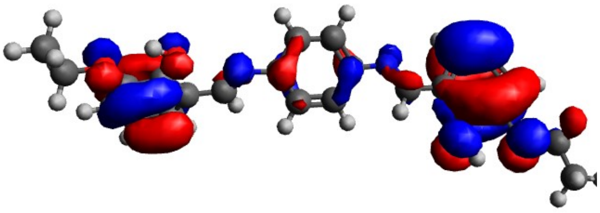

HOMO -2

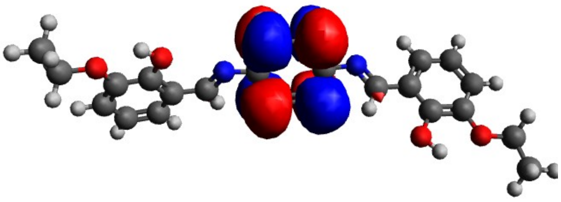

LUMO +2

(3b)

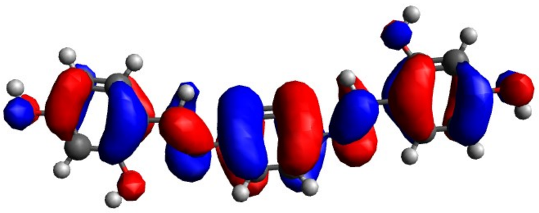

HOMO

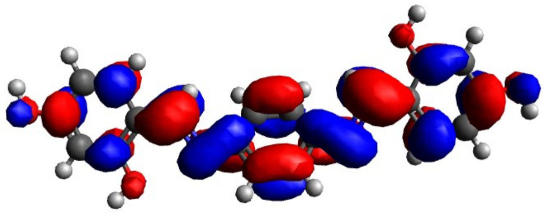

LUMO

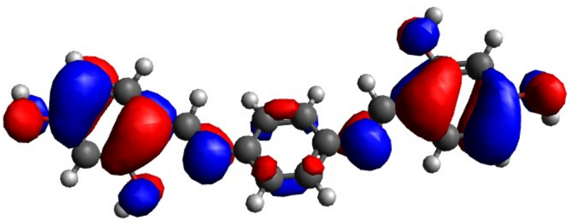

HOMO -1

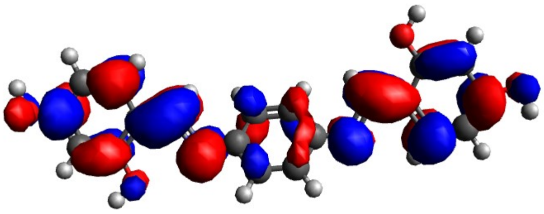

LUMO +1

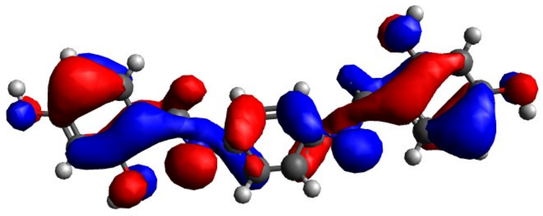

HOMO -2

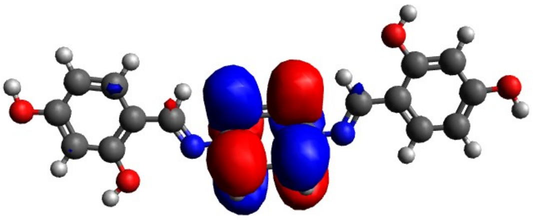

LUMO +2

(3c)

Figure S11: The MO surfaces of **3a-3c**

Table S11: Cartesian coordinates of **3c** compound.

Atom	X-axis	Y-axis	Z-axis
C	-0.70891	0.194841	0.715019
C	0.675732	0.230824	0.715565
C	1.415265	-0.73314	0.023314
C	0.725912	-1.76124	-0.62836
C	-0.6536	-1.79609	-0.62976
C	-1.39646	-0.80534	0.021195
H	-1.26452	0.926939	1.294091
H	1.192898	0.990609	1.294728
H	1.303507	-2.52901	-1.13439
H	-1.18985	-2.5922	-1.13766
N	-2.79105	-0.91421	-0.00276
N	2.813734	-0.76859	0.007152
C	3.458691	0.327179	-0.08126
H	2.925744	1.285981	-0.20534
C	-3.50793	0.140965	-0.03699
H	-3.0609	1.141791	-0.10731
C	-4.96439	0.091027	-0.0049
C	-5.64402	-1.12529	0.11108
C	-5.71568	1.274331	-0.09305
C	-7.0233	-1.18037	0.138096
H	-5.0461	-2.02877	0.178504
C	-7.10711	1.220997	-0.0642
C	-7.7535	0.002583	0.049787
H	-7.67658	2.144285	-0.13315

H	-8.83848	-0.02345	0.070431
C	4.907235	0.477992	-0.05965
C	5.833434	-0.57048	0.1163
C	5.408174	1.775414	-0.22784
C	7.19827	-0.28982	0.113287
C	6.761666	2.051769	-0.22907
H	4.692145	2.582568	-0.36124
C	7.659108	1.004295	-0.05714
H	7.900988	-1.10878	0.248494
H	8.72794	1.193225	-0.05438
O	-5.04698	2.441549	-0.20514
H	-5.6695	3.174856	-0.25119
O	5.385803	-1.82639	0.286512
H	6.138842	-2.41692	0.401776
H	-7.53321	-2.13336	0.228217
H	7.116101	3.06778	-0.36186

Table S12: Cartesian coordinates of **3e** compound.

Atom	X-axis	Y-axis	Z-axis
C	-0.7553	0.191977	-0.60908
C	0.611844	0.397599	-0.53493
C	1.470136	-0.63908	-0.15523
C	0.915147	-1.88383	0.159607
C	-0.44391	-2.09834	0.050755
C	-1.30609	-1.05845	-0.31251
H	-1.40449	1.004351	-0.92365
H	1.020977	1.380029	-0.75387
H	1.582596	-2.68293	0.468132
H	-0.87286	-3.07225	0.267276
N	-2.67418	-1.33835	-0.39421
N	2.855525	-0.48828	-0.03766
C	3.478291	0.215548	-0.89862
H	2.946644	0.651345	-1.76211
C	-3.52261	-0.45056	-0.04788
H	-3.20946	0.513589	0.375935

C	-4.95951	-0.65611	-0.17228
C	-5.48809	-1.83503	-0.7225
C	-5.83651	0.336371	0.262167
C	-6.84743	-2.01133	-0.83204
H	-4.79136	-2.59663	-1.05596
C	-7.22645	0.151912	0.145367
C	-7.72865	-1.01475	-0.39744
H	-8.79853	-1.16308	-0.48945
C	4.902968	0.518146	-0.88996
C	5.801654	0.091747	0.093722
C	5.395372	1.29715	-1.95288
C	7.160302	0.450783	-0.00294
C	6.72413	1.642691	-2.03985
H	4.694592	1.626446	-2.715
C	7.619544	1.217515	-1.05684
H	8.666724	1.489346	-1.12386
O	-5.36641	1.480408	0.796275
H	-6.1277	2.031191	1.027582
O	5.411801	-0.65502	1.13822
H	6.19977	-0.81856	1.67774
H	-7.24936	-2.92449	-1.25782
H	7.083457	2.244307	-2.86736
O	7.91103	-0.02928	1.01976
O	-7.94852	1.201904	0.607342
C	-9.36989	1.135457	0.536715
H	-9.72045	0.26469	1.107734
H	-9.67417	0.998332	-0.51011
C	-9.9135	2.416755	1.102182
H	-11.0055	2.409324	1.067674
H	-9.60206	2.541951	2.142762
H	-9.55601	3.274744	0.526389
C	9.302608	0.273948	1.050032
H	9.777632	-0.11301	0.138066
H	9.435266	1.36451	1.064221
C	9.879844	-0.36417	2.281584

H	9.739466	-1.44819	2.256373
H	10.95064	-0.15659	2.345501
H	9.398014	0.028299	3.181176

Table S13 Cartesian coordinates of **3g** compound.

Atom	X-axis	Y-axis	Z-axis
C	-0.70365	0.124003	0.715911
C	0.681714	0.134914	0.717512
C	1.406103	-0.83733	0.020303
C	0.696529	-1.84866	-0.63676
C	-0.68358	-1.85865	-0.63916
C	-1.41076	-0.85866	0.016082
H	-1.24588	0.863027	1.299126
H	1.211514	0.882144	1.301828
H	1.260006	-2.62382	-1.14779
H	-1.23325	-2.64193	-1.15299
N	-2.80689	-0.93932	-0.01074
N	2.803313	-0.89719	0.003599
C	3.466426	0.190822	-0.07338
H	2.94521	1.157487	-0.19017
C	-3.4993	0.134606	-0.04356
H	-3.02416	1.12294	-0.11311
C	-4.95063	0.129893	-0.01213
C	-5.67355	-1.06106	0.107081
C	-5.67522	1.332728	-0.10563
C	-7.05033	-1.08315	0.132472
H	-5.10821	-1.98487	0.179368
C	-7.06262	1.325821	-0.08093
C	-7.7494	0.123641	0.036419
C	4.911109	0.326993	-0.04816
C	5.835806	-0.72842	0.121737
C	5.432461	1.617296	-0.20622
C	7.198787	-0.46691	0.12219
C	6.784023	1.888061	-0.20542
H	4.729933	2.436675	-0.335

C	7.675973	0.828457	-0.04002
O	-4.97599	2.479731	-0.21991
H	-5.57686	3.230812	-0.26865
O	5.377675	-1.98003	0.283838
H	6.124103	-2.57951	0.396002
H	-7.59396	-2.01875	0.226808
H	7.153307	2.901654	-0.32996
H	7.912073	-1.27704	0.250634
H	-7.62536	2.25223	-0.15431
O	-9.0938	0.184123	0.052654
H	-9.46178	-0.7028	0.131136
O	9.010998	0.994388	-0.02727
H	9.227852	1.925239	-0.14744
